# Parameter Inference for Computational Cognitive Models with Approximate Bayesian Computation

**DOI:** 10.1111/cogs.12738

**Published:** 2019-06-03

**Authors:** Antti Kangasrääsiö, Jussi P. P. Jokinen, Antti Oulasvirta, Andrew Howes, Samuel Kaski

**Affiliations:** ^1^ Department of Computer Science Aalto University; ^2^ Department of Communications and Networking Aalto University; ^3^ School of Computer Science University of Birmingham

**Keywords:** Parameter estimation, Inference, Cognitive models, Machine learning, Computational statistics, Bayesian optimization, Approximate Bayesian computation

## Abstract

This paper addresses a common challenge with computational cognitive models: identifying parameter values that are both theoretically plausible and generate predictions that match well with empirical data. While computational models can offer deep explanations of cognition, they are computationally complex and often out of reach of traditional parameter fitting methods. Weak methodology may lead to premature rejection of valid models or to acceptance of models that might otherwise be falsified. Mathematically robust fitting methods are, therefore, essential to the progress of computational modeling in cognitive science. In this article, we investigate the capability and role of modern fitting methods—including Bayesian optimization and approximate Bayesian computation—and contrast them to some more commonly used methods: grid search and Nelder–Mead optimization. Our investigation consists of a reanalysis of the fitting of two previous computational models: an Adaptive Control of Thought—Rational model of skill acquisition and a computational rationality model of visual search. The results contrast the efficiency and informativeness of the methods. A key advantage of the Bayesian methods is the ability to estimate the uncertainty of fitted parameter values. We conclude that approximate Bayesian computation is (a) efficient, (b) informative, and (c) offers a path to reproducible results.

## 1 Introduction

*Computational cognitive models* are computer programs used for theorizing and explaining cognitive processes, and explaining their behavioral consequences (Anderson et al., 2004; Botvinick, Braver, Barch, Carter, & Cohen, 2001; Busemeyer & Townsend, 1993; Daw, O'doherty, Dayan, Seymour, & Dolan, 2006; Eliasmith et al., 2012; Geisler, 2011; Howes, Lewis, & Vera, 2009; Tenenbaum, Kemp, Griffiths, & Goodman, 2011). They take many forms, including production systems architectures (Anderson et al., 2004; Howes et al., 2009), reinforcement learning models (Daw et al., 2006), optimal control models (Geisler, 2011), and neural network and hybrid models (Botvinick et al., 2001; Busemeyer & Townsend, 1993; Eliasmith et al., 2012). Interest in this class of models is motivated by the precision and expressiveness that computer programs offer for modeling the human mind. They permit seeking a strong correspondence between theoretical assumptions and empirical observations of human behavior, showing promise in being able to model complex emergent phenomena that may be out‐of‐reach for fully analytic models. However, the increasing complexity of models introduces a new problem: *parameter inference*. The correspondence between model predictions and real observations, or *model fit*, is often taken as an indicator of progress in modeling: a new model is credible, if it predicts important empirical observations more accurately than an existing model, assuming, in addition, that key assumptions of the model are theoretically plausible.

The model fit is determined jointly by the model structure, and by the values of the model variables. Some variable values have been chosen prior to empirical observations, and have their place, for example, as theoretical postulates. Others, called here *parameters*, are determined from empirical data, and can take almost any value depending on the situation. For example, the response criterion parameter β in signal detection theory (Macmillan, 2002) relates to how conservative a decision‐maker is, and depends on idiosyncratic and task‐specific factors, as well as the experimental task. In addition, there are parameters that are fairly stable and have well‐known limits. For example, the parameter value that governs the duration of saccades as a linear function of the visual angle is well‐known in eye movement models (Abel, Troost, & Dell'Osso, 1983; van Beers, 2007). The *parameter inference problem* is to determine the parameter values for a specific model, given empirical observations.

When selecting a parameter inference method, a researcher faces multiple decisions (Myung & Pitt, 2016). The choices affect the quality, efficiency, and reproducibility of research. First, the size of the parameter search space, taking into account both the number of parameters and their plausible ranges, must be considered. On one hand, if the space is too large, searching for the optimal parameter values might require more time and computational resources than necessary. On the other hand, if the space is too small, it might exclude the optimal parameter values and thus lead to worse model fit. Second, once the parameter search space is defined, one needs to choose the method for searching through this space; that is, the inference method. Different commonly used methods and their trade‐offs are discussed later in this paper. After inference, various analyses can still be performed. For example, the model predictions can be validated against hold‐out data to guarantee that the model has not been over‐fit to the specific set of training data. Also, the sensitivity of the model fit to changes in the parameter values can be estimated, to check that minor changes to the parameter values do not lead to large changes in predictions (Ratcliff, 2013). Some inference methods might provide these analyses as side products of the inference process, while other methods do not. Finally, if the various decisions made regarding the parameter inference process are not documented appropriately then there is another reason that the results, and the modeling process, are difficult to replicate.

Although parameter inference has been a part of cognitive science research for many years, there are multiple occasions where either the methods have been suspect in terms of their capability or the reporting of the inference process lacks sufficient detail. Recent introductions to model comparison, fitting, and parameter selection are part of an effort to improve the state of affairs (Farrell & Lewandowsky, 2018; Myung & Pitt, 2016; Turner, Dennis, & Van Zandt, 2013; Turner & Van Zandt, 2012). Especially in the case of complex simulation models of cognition, where likelihood functions are difficult or impossible to establish, recent work on likelihood‐free methods has shown promise (Turner, Sederberg, & McClelland, 2016; Turner & Van Zandt, 2018). However, the full potential of such methods has not been demonstrated with worked and critically assessed examples of complex cognitive simulation models. To this end, this paper builds and contributes to this work by investigating the use of Bayesian optimization (BO) and approximate Bayesian computation (ABC) to infer parameters of computational cognitive models. This is important because the modeling field has not established commonly agreed standards for parameter inference.

At least four issues related to parameter inference can be identified in the recent computational cognitive science literature. First, parameter values can be set based on external sources in the prior literature without considering how changing these parameters might affect the results. This is often justified in case of parameters that are assumed to be very general, and that should therefore not vary between tasks. The issue, however, is that external sources may have involved very different experimental methods, samples, and modeling aims, all of which may make parameters incompatible for a completely new model. Thus, although values from external sources can serve as prior information that indicate probable ranges for model parameter values, the reliability of these prior values should also be taken into account.

Second, sometimes parameter values are used that are unlikely given prior work, but that happen to lead to adequate model fits to the new data. This might suggest that these unlikely parameter values are compensating for inadequacies in the model structure, or it might be that the model is just very insensitive to the values of those parameters. This issue is complementary to the first; here, the prior knowledge is ignored, whereas in the first issue the prior knowledge is adopted blindly. Third, the sensitivity of the model fit either to changes in the random seed number or perturbations to parameter values is not analyzed, or at least not reported. Fourth, some papers only report that the parameters “were fit to data,” without reference to the fitting procedure. This makes the research challenging to replicate as another inference method might well lead to different parameter values. It is challenging to draw conclusions about the reliability of parameter values inferred in a previous study without sufficient details of the process that lead to the particular values being chosen. We hypothesize that these issues might be caused partly by the unavailability of efficient, critically tested, and openly available methods for performing parameter inference for computational cognitive models, and partly by the fact that the researchers are unfamiliar with the full spectrum of methods and practices that are available.

The main goal of this paper is to demonstrate that principled and well‐documented parameter inference is possible even with some of the most sophisticated simulation models in cognitive science. The methods that we investigate are applicable to most computational cognitive models and allow inference of some tens of parameters at a time. The methods promise efficiency compared to other commonly used methods, and with parallelization may be able to handle computationally very expensive models. The methods are also designed to provide principled probabilistic estimates of the model fit across the parameter search space, thus facilitating sensitivity analysis. Also, open‐source implementations of the methods are publicly available (Lintusaari et al., 2017). Our goal, then, is to investigate these new fitting methods with the aim of helping to lower the barrier of applying them in cognitive science.

The first of the new methods—a method that is already being adopted in cognitive science—is BO, which is a sample‐efficient algorithm for global optimization of stochastic functions. The second method is BO combined with ABC, which enables the inference of an approximate posterior distribution for the model parameters, thus providing an intuitive quantification of acquired knowledge of the best parameter values given the available observation data. We restrict the scope of this paper to generally applicable, model‐agnostic, inference methods that are capable of operating with models that generate predictions but do not have a tractable likelihood function.

We evaluate the models against three criteria. The first criterion is computational efficiency. We quantify efficiency as the amount of computational resource required to achieve a certain predetermined level of model fit. We measure computational resources with CPU‐hours^1^ and model fit by prediction error on a separate validation dataset. The efficiency in parameter inference is crucial in model development, as it enables more iterations of evaluation. It is especially important when working with computationally expensive cognitive models, as each prediction with new parameter values might take hours to complete.

The second criterion is informativeness: how much information do we gain about the model fit with different parameter values within the feasible parameter space. Different methods provide different types of characterizations of the model fit: some only provide a point estimate, while others provide probabilistic estimates over the whole parameter space. The quantification of the model fit over the parameter space is important in many ways, for instance, allowing a principled estimation of the reliability of the inference results. In addition, the quantification of fit is informative for subsequent selection of reasonable bounds on the parameter space, thereby supporting a more focused search of the feasible parameter regions. Lastly, the quantification provides insight into model development, for example, by pointing out that a certain parameter might not meaningfully affect the model fit. The third criterion is replication. Reproducible results are crucial to the progress of science. Meeting efficiency and informativeness criterion should, in addition, support replication. Replication may be supported, for example, by methods that quantify the model fit.

The paper is structured as follows. We first outline the scientific quantitative modeling process, and discuss the common practices used for fitting cognitive models. We then discuss considerations general for inference with simulator models, and then explain how BO and ABC can be applied in this context. Finally, we present case examples using two different simulator model types from recent cognitive science literature. The first is an Adaptive Control of Thought—Rational (ACT‐R) model that predicts how learning affects the duration of different cognitive stages in solving a mathematical problem. The ACT‐R cognitive architecture contains a large number of parameters, which often need to be adjusted between experiments. The proposed methods are promising for parameter estimation in ACT‐R, where simulations are often computationally intensive and do not permit the computation of a likelihood. The second case is a model that predicts eye movements and task completion time when searching for a target item from a visually displayed computer drop‐down menu (Chen, Bailly, Brumby, Oulasvirta, & Howes, 2015). The model uses reinforcement learning (RL) to compute the optimal, utility‐maximizing policy. The optimal policy depends on the initial parameters of the model, and because training the model is computationally intensive, it provides an interesting case study for our approach to parameter inference.

## 2 Background

Computational cognitive models are used widely throughout the cognitive sciences. They are built to simulate human cognition through the stepwise execution of a program. Like other models, cognitive models map model parameters to predictions of cognitive behavior. Parameters can represent stable aspects such as memory capacities or situationally changing aspects like task goals. It is common that these models include stochastic elements, which means that they map parameters to not to a single prediction, but to a *distribution of predictions*. This means that when the model is executed multiple times, the predictions may be different, even if the parameters are kept fixed.

Computational cognitive modeling is one instance of a class of simulative modeling methods used across the sciences. The focus in this section is on outlining common methods that are used for fitting such models to data. Quantitative modeling, a central activity in science generally, may involve following activities:

- **Model design:** How should the model be formulated? This consists of writing down the formulas and algorithms that define a parametric model *M* with free parameters θ and possibly also a prior probability distribution *P*(θ) which quantifies how likely different parameter values are *a priori*.
- **Experiment design:** How should one collect the observation data *D*_*obs*_ for evaluating the quality of one or multiple different models? This consists of estimating the amount and quality of data that need to be collected and coming up with suitable data collecting procedures.
- **Parameter inference:** Given a set of observation data and a model, which parameter values offer the best model fit? This consists of optimizing a function that quantifies the model fit. For example, the expected prediction error, observation likelihood *P*(*D*_*obs*_ *|*θ), and posterior probability *P*(θ*|D*_*obs*_) = *P*(*D*_*obs*_ *|*θ)*P*(θ)*/P*(*D*_*obs*_) are commonly used.^2^
- **Model evaluation:** How well is a model able to reproduce the phenomena of interest? This is generally done by first generating a set of predictions from the model. Monte Carlo sampling is commonly used: *D*_*pred*_ ∼ *P*(*D|*θ).^3^ This sampling can be done either just using the optimal point estimate, θ* = *θ*, or, for example, using samples from the posterior distribution, θ ∼ *P*(θ*|D*_*obs*_). This set of predictions may then be compared to a separate set of validation data *D*_*val*_, which was not used in any way during the inference.
- **Model selection:** Which of multiple models is the most credible explanation for the phenomena we are interested in? This generally consists of comparing the fit of multiple distinct models and selecting the best one according to a suitable criterion. For example, average prediction error or the Bayes factor *P*(*D|M*_1_)*/P*(*D|M*_2_) can be used.

Model construction, for instance, related to higher order cognition, has been discussed by Cooper (2002), experiments from the point‐of‐view of model comparison by Myung and Pitt (2009), and evaluation and comparison of models by Myung, Tang, and Pitt (2009) and Howes et al. (2009) among others.

This paper focuses specifically on parameter inference for cognitive models, which is a topic that has received increasing interest recently. Papers have pointed out different issues with the parameter inference methods used with cognitive models. One issue that has been raised is the fact that manual parameter tuning is still commonly used instead of automatic parameter inference methods (Lane & Gobet, 2013; Raymond, Fornberg, Buck‐Gengler, Healy, & Bourne, 2008; Said, Engelhart, Kirches, Körkel, & Holt, 2016). Another issue has been the fact that the most basic automatic inference methods might either not be efficient enough, or be unable to visualize the model fit over the parameter space (Gluck, Stanley, Moore, Reitter, & Halbrügge, 2010; Moore, 2011).

Although many of these papers also propose various alternative methods for dealing with these issues, such as the adaptive mesh refinement approach (Moore, 2011), these approaches tend to lack rigorous theoretical justification and analysis, and performance might only be evaluated using a single example case. In general, it would be preferable to use principled approaches that are well motivated from a theoretical standpoint, and even offer general performance guarantees. Also, some of these approaches, such as mathematical programming (Said et al., 2016), require altering or rewriting the model to match the assumptions of the inference method. It would be preferable to have approaches that are generally applicable for any computational cognitive model, such that the inference procedure does not force limitations to the model structure, and that alterations to the model do not necessitate alterations to the inference procedures as well. There is also currently significant variability in the parameter inference practices used in the field. While in some cases the parameter inference process is thoroughly documented and a careful analysis is done of the remaining uncertainty in the parameters (e.g., Burling & Yoshida, 2017), there are also cases where the precise method used for parameter inference is not discussed, and the inferred parameters are reported just as a point estimate without any discussion of their uncertainty or sensitivity.

A cognitive model is essentially an algorithm that is computed in discrete steps. Technically speaking, at every step *t*, a stochastic function *f* is used to generate new values for the model state variables based on the current state: *s*_*t*_ **∼ *f* (*s*_*t−*1_, *t*, θ). The function *f* has free parameters θ, and may contain complex operations, such as numerical optimization or Monte Carlo sampling. The state *s* may also be composed of a large number of variables, such as a description of a person's short‐term memory content. A benefit of expressing theories computationally instead of verbally is that the former allow higher representational power and specificity. A large number of alternative assumptions can be conveniently investigated, as often only changes to the model parameters, or minor changes to the model implementation source code, are required. Additionally, these models can predict not only behavioral outcomes, but also the intermediate (often unobserved) cognitive steps.

Parameter inference is often challenging with computational cognitive models. This is due to the fact that it is often not possible to solve the optimal parameter values directly, as the models are stepwise executed programs instead of a set of analytic equations. Generally, the only way to evaluate the model fit of certain parameter values is to execute the model using these values, and to then evaluate the quality of the resulting predictions against the observation data. This process is similar to manual fitting, although automatic methods are able to apply efficient numerical optimization methods and parallelism for greatly improving the efficiency and reliability of this approach. In the next section, we discuss different methods for performing this inference for computational cognitive models.

## 3 Probabilistic inference for computational cognitive models

### 3.1 Traditional fitting methods

#### 3.1.1 Manual fitting

Perhaps the most rudimentary way to search for appropriate parameters is to select values manually and compare the simulation results visually with the observation data. A benefit of this method is that it requires no additional software implementations except for the model itself, and a way to visualize the resulting predictions. For this reason, it is often one of the first approaches to parameter tuning that is used when developing models, and might be useful as a model debugging tool in general.

However, when used as the primary method for parameter inference, this approach has many drawbacks. First, it is very labor‐intensive, requiring the researcher to manually go through a possibly large number of parameter combinations. Second, the approach has no guarantees for optimality of the parameters or time it takes for the process to converge. Third, it brings in multiple possible biases that individual researchers may have, thus making the inference process difficult to replicate, as researchers may have different opinions on what constitutes a good model fit.

As an example of manual fitting, consider the model of movement planning by Harris and Wolpert (1998). Most of the model parameters were set to values adopted from the literature, while the rest were set manually. For example, the post‐movement fixation period was set to 50 ms without a stated reason. Also, further manual adjustment was done case‐by‐case, as an additional cost of 10 ms was added to eye movements in some of the model variants but not in others. Samuelson, Smith, Perry, and Spencer (2011) present a model for word learning where six parameters were tuned manually, while the rest of the model parameters were adopted directly from an earlier study, where all of the parameters were tuned manually (Faubel & Schöner, 2008). With many ACT‐R models (Altmann & Trafton, 2002; Gonzalez, Lerch, & Lebiere, 2003; Lewis & Vasishth, 2005) parameters are either set to values adopted directly from earlier literature or set manually. Chen et al. (2015) presented a cognitive model of visual search that used reinforcement learning to discover bounded optimal strategies of eye movements. Parameters of the model were set manually based on existing literature. For example, the duration of eye fixations was fixed to 400 ms based on previous research on the investigated task. For comparison, Kangasrääsiö et al. (2017) optimized the value of the same parameter in a later study using automatic methods, ending up with a smaller fixation duration around 250 ms, which resulted in a better model fit to observation data.

#### 3.1.2 Grid search

Grid search, also known as brute‐force search, works by dividing the parameter space to a large number of small regions (called cells), often by using an even grid. For each cell, predictions are generated using parameter values from the corresponding parameter region. Finally, the parameter values that yielded the best model fit are selected.

A benefit of this method is that it is very simple to implement, often requiring only few lines of additional code to iterate through the grid and keep track of the best parameters so far. With a sufficiently dense grid, this method also gives some guarantee of global optimality, as it literally searches through the entire parameter space for the optimal values. As the method evaluates the model fit all around the parameter space, it enables the model fit to be easily visualized as well. This method is also parallelizable, as given sufficient computational resources we might even simulate at all of the grid cells at the same time.

However, a drawback of this method is the generally poor efficiency. First, it does not use the past simulations to its advantage, which might lead to a large number of simulations with parameters that effectively are already known to be non‐optimal. Second, the number of samples needed is *n* ^*d*^, where *d* is the number of parameters (dimensionality of search space) and *n* is the number of grid cells per dimension. This means that inferring multiple parameters simultaneously (large *d*) or using a dense grid (large *n*) might make this method computationally very expensive.

There are multiple recent examples of grid search being used for optimizing parameter values and characterizing model fit across the parameter space. We here list few as typical examples: Blouw, Solodkin, Thagard, and Eliasmith (2016) used grid search for fitting one parameter of a semantic categorization model. Lee, Betts, and Anderson (2016) used grid search for fitting two parameters of a memory model. Patil, Hanne, Burchert, De Bleser, and Vasishth (2016) fit multiple parameters for an ACT‐R model for sentence processing using brute‐force search. Stocco (2017) compared two competing action selection models, both using the ACT‐R cognitive architecture, and used a thoroughly reported grid search to fit the parameters. Honda, Matsuka, and Ueda (2017) modeled binary choice in terms of attribute substitution in heuristic use, and fitted one parameter with grid search. There are also examples of heuristic extensions to grid search for improving the scalability. One such was presented by Godwin, Reichle, and Menneer (2017), who modeled fixation revisits in visual search, and used successively more constrained grid searches to fit six model parameters. Each successive grid search centered on the best‐fitting parameter values of the previous search, using smaller cell sizes.

#### 3.1.3 Local search

Local search, a widely used approach for finding the minimum of approximately continuous functions, starts from a random location within the parameter search space and then moves to the direction that seems to locally lead to a better model fit. Once the method converges, meaning that no small changes in the parameter values lead to any further improvement, the method has found a local optimum. When the model fit surface is unimodal, meaning that it has only one clear optimum, this method is often efficient in finding that optimum. Another benefit of this method is also generally good scalability to large parameter spaces, as the method only needs to make evaluations along a path to the optimum, instead of all across the parameter space.

However, there are certain notable drawbacks as well. If the optimized function might have multiple local optima, the method generally needs to be restarted multiple times from different initial locations to increase the probability that the global optimum is eventually found, which increases the overall computational cost. Also, local search generally works less efficiently when the optimized function is stochastic, as this makes it difficult to estimate the gradient of the model fit.^4^ Although this may be alleviated by averaging multiple model evaluations, this will again increase the computational cost. A third drawback of this method is that it does not provide any insights about the global model fit surface, which means that further analysis is required to quantify the reliability of the results. A fourth drawback is the difficulty of parallelization, as the approach specifically requires sequential model evaluations. In general, the only viable approach to parallelization is to execute multiple independent local optimization processes in parallel.

There are multiple algorithms for local search, designed with different limitations in mind. With computational cognitive models, four limitations stand out:

1. No easy access to the gradient of the optimized function (often the likelihood function *P*(*D*_*obs*_|θ)).
2. Stochastic model evaluations, meaning that repeated evaluations with the same parameters may lead to different model fits.
3. Non‐continuity of the model fit, meaning that in some regions of the parameter space small changes in the parameter values may lead to large changes in the model fit.
4. Computationally expensive model evaluations, meaning that one should aim for a minimal number of predictions to be generated from the model during inference.

These limitations generally imply that derivative‐free local optimization methods (Rios & Sahinidis, 2013) need to be used. Although one could hope that the gradient could be estimated from samples, this is often challenging in practice due to the combined effect of the second, third, and fourth limitations mentioned above.

One of the most widely used derivative‐free methods is Nelder–Mead simplex optimization (Lagarias, Reeds, Wright, & Wright, 1998).^5^ One main benefit of Nelder–Mead over other derivative‐free methods is its wide availability: for example, the MATLAB function fminsearch implements this and uses it as a default, and it is often available in many other optimization packages as well. There are multiple examples of Nelder–Mead being used over the years, as well as recently. Bogacz, Brown, Moehlis, Holmes, and Cohen (2006) optimized the cost function of a choice model to empirical data using subplex (Rowan, 1990), a variant of Nelder–Mead. Vandekerckhove and Tuerlinckx (2007) optimized the parameters of the Ratcliff diffusion model using Nelder–Mead. Said et al. (2016) optimized the performance of an ACT‐R model with different methods, and Nelder–Mead was one of the best‐performing methods. Logačev and Vasishth (2016) optimized the parameters of a sentence parsing model using Nelder–Mead. Gagliardi, Feldman, and Lidz (2017) optimized the parameters of a language learning model using the fminsearch. For situations where the optimized function has multiple optima, a combination of Nelder–Mead optimization and grid search has been used. For example, Wallsten, Pleskac, and Lejuez (2005), as well as Glöckner and Pachur (2012) used grid search to provide starting values as inputs to the Nelder–Mead method, in order to combat the problem of highly irregular surfaces and the subsequent risk of getting stuck in a local optimum.

### 3.2 Bayesian optimization

Bayesian optimization (Brochu, Cora, & De Freitas, 2010) is a popular modern approach to global optimization. One core idea of the method is to use a *surrogate model* for approximating the model fit across the parameter space. Another core idea is to use an *acquisition rule* for selecting which parameter values are used for generating predictions, based on the surrogate model. Inference is performed through a sequence of optimization rounds. At the beginning of each round, the acquisition rule is used to select a set of parameter values that will be used to generate predictions. The locations are balanced such that they cover both unknown regions of the parameter space (exploration) and regions with high probability to lead to a good model fit (exploitation). After predictions have been generated at each location, the surrogate model is updated based on the observed model fits, and the next optimization round begins. The final parameter estimates are often chosen to be the parameter values that lead to the best predicted model fit on average.

Gaussian process (GP) regression models (Rasmussen, 2004) are by far the most commonly used family of surrogate models used in BO. This is because of the high flexibility of the model family, which gives it the capacity to approximate a large subset of model fit surfaces that are encountered in practice. GP models are also able to model the stochasticity of model fit, thus allowing a principled estimation of its mean and variance everywhere in the parameter search space.

Common acquisition rules include the probability of improvement rule (PI), the expected improvement rule (EI), the entropy search rule (ES), and the upper confidence bound rule (UCB) (Snoek, Larochelle, & Adams, 2012). They all aim to find the optimal value, but with slightly different criteria. PI maximizes the probability of finding a value better than current optimum, EI maximizes the magnitude of improvement over current optimum, ES maximizes the information about the location of the global optimum, and UCB optimizes the balance between exploration and exploitation.

Bayesian optimization has been shown to be sample efficient (e.g., Snoek et al., 2012), which means that good parameter estimates are generally available after relatively few predictions from the model. Intuitively, this is because the global surrogate allows the acquisition rule to avoid regions where the optimum is unlikely to be in. An additional benefit for further analysis of model performance is that the surrogate generated in the process is a good characterization of the global model fit as a function of the parameter values. Also, the sampling process for BO is easy to parallelize, allowing large batches to be computed simultaneously in a computing cluster.

One drawback of BO is the need to specify values for multiple hyperparameters, which determine how fast the model fit is expected to change and how noisy the evaluations are expected to be. These values can be chosen either based on our prior knowledge or they can be estimated based on initial tests. Further, the values can be further adjusted during the optimization process, based on the observed samples. However, as exhaustive search over all possible hyperparameter values might not be feasible in practice, it is common that some “artistry” is required in setting good initial values, based on prior experience with GP models and BO. Another drawback is that updating the surrogate model and evaluating the acquisition rule also require computational resources. In many cases, this inference overhead might not be an issue, but it may become noticeable if the function being optimized happens to be cheap to evaluate, or if the parameter space is high‐dimensional and a large number of samples are used. However, there are also extensions of BO that help with specific issues. For example, it is possible to perform optimization in high‐dimensional spaces when certain general assumptions hold (e.g., Wang et al., 2013).

Bayesian optimization has not yet been widely used with computational cognitive models. Turner et al. (2013) used BO to fit parameters to cognitive memory models, and Turner et al. (2016) used Bayesian analysis to formally compare simulation‐based neurological models. Kangasrääsiö et al. (2017) used BO to find the parameter values of a cognitive model of visual search in menus. Further, this approach has been used recently in other fields, for example, for parameter tuning biomedical models (Ghassemi, Lehman, Snoek, & Nemati, 2014) and speech recognition models (Watanabe & Le Roux, 2014). It has also been used for fMRI study design (Lorenz et al., 2016) and optimization of game engagement (Khajah, Roads, Lindsey, Liu, & Mozer, 2016). Lee and Wagenmakers (2014) present a guide to Bayesian cognitive modeling with practical examples such as estimating coefficient of agreement in a decision‐making task. What is missing are worked examples of using BO and ABC, introduced below, in parameter inference of complex cognitive simulation models. This is necessary for introducing these techniques to a larger cognitive scientific audience that works with complex simulation models.

### 3.3 Approximate Bayesian computation

Approximate Bayesian computation (Sunnåker et al., 2013) is a method that allows estimating posterior probability distributions for the parameters of computational cognitive models. The idea of ABC is that predictions made with various different parameter values can be used to construct an approximation of the observation likelihood function for the model, which can then be used to estimate the posterior distribution. ABC has been used earlier for computational cognitive models. Turner and Van Zandt (2012) demonstrate the use of ABC for estimating the parameter values of cognitive simulation models, such as a memory retrieval model. Turner and Sederberg (2014) introduce the probability density approximation method—a likelihood‐free method for posterior estimation—and demonstrate its use with simulations of various tasks, such as signal detection and decision‐making tasks. ABC has been used in various other fields as well, especially for inference with complex simulator models (Csilléry, Blum, Gaggiotti, & François, 2010). A recent review of various ABC methods was made by Lintusaari, Gutmann, Dutta, Kaski, and Corander (2017).

The idea of ABC is not very involved, but requires a short mathematical explanation. First, as a reminder, Bayesian inference is based on the assumption that our knowledge of what parameter values are correct is described by a probability distribution. Our prior knowledge of likely values is described by the prior distribution *P*(θ). After observations *D*_*obs*_ are made, the observation likelihood function *P*(*D*_*obs*_|θ) is combined with the prior to update our knowledge of the probability of different parameter values. Our updated state of knowledge is the posterior distribution *P*(θ|*D*_*obs*_), which is computed from the Bayes’ formula *P*(θ*|D*_*obs*_) = *P*(*D*_*obs*_ *|*θ) *P*(θ)*/P*(*D*_*obs*_). In this inference framework, the observation likelihood *P*(*D|*θ) is determined by the structure of the assumed cognitive model. However, the problem with computational cognitive models is that the model structure, when expressed as an explicit mathematical formula for *P*(*D|*θ), can be very complicated. This means that in practice, the likelihood function cannot be evaluated during inference.

The key insight of ABC is that it is possible to avoid evaluating the likelihood, if one is satisfied with an approximation of the posterior distribution. First, assume a function for computing the model fit, which in ABC in called the *discrepancy function*
$\delta (D_{\mathrm{obs}},D_{\mathrm{pred}}\to \left[0,\infty \right).$Using δ, one can define a random variable

$$d_{\theta }:=\delta \left(D_{\mathrm{obs}},D_{\mathrm{pred}}\right)|D_{\mathrm{pred}}∼P\left(D|\theta \right).$$

The distribution of *d*_θ_ quantifies precisely the distribution of model fits each θ is associated with. If the discrepancy function *δ* has the property that δ(*D*_*obs*_, *D*_*pred*_) = 0 ⇔ *D*_*obs*_ **= *D*_*pred*_, we can write the likelihood as

$$\begin{array}{cc} P(D_{\mathrm{obs}}|\theta ) & =P(D_{\mathrm{obs}}=D_{\mathrm{pred}}|D_{\mathrm{pred}}∼P\left(D|\theta \right))\\ & =P(\delta \left(D_{\mathrm{obs}},D_{\mathrm{pred}}\right)=0|D_{\mathrm{pred}}∼P\left(D|\theta \right)\\ & =P(d\theta =0|\theta ). \end{array}$$

The first equality is the definition of the likelihood function, the second uses the assumed property of δ, and the third uses the definition of *d*_θ_. Now the ABC approximation is to simply relax this strict requirement by

$${\tilde{P}}_{\varepsilon }\left(D_{\mathrm{obs}}|\theta \right)=P\left(d_{\theta }<\varepsilon |\theta \right),$$

where ε ∈ [0, *∞*) is a chosen approximation threshold. ${\tilde{P}}_{\varepsilon }\left(D_{\mathrm{obs}}|\theta \right)$ is called the ε‐approximate likelihood, and when combined with the prior, can be used to compute the corresponding ε‐approximate posterior.

In practice, the ε‐approximate likelihood is constructed by making a set of predictions with various different parameter values. This allows one to estimate the distribution of *d*_θ_ across the parameter space, which then leads directly to the ε‐approximate likelihood. Different approaches to ABC generally differ in how they construct the estimate for *d*_θ_. The earliest methods used a brute‐force approach, where predictions were made densely all across the parameter space. A recent method proposed by Gutmann and Corander (2016), which we use in this paper as well, is to use BO for constructing a GP estimate for *d*_θ_. Technically, as the marginal distribution of a GP in each point θ is a Gaussian distribution *N*(μ(θ)*,* σ(θ)), we can further approximate

$$P\left(d_{\theta }<\varepsilon |\theta \right)\approx \Phi \left(\varepsilon |\mu \left(\theta \right),\;\sigma \left(\theta \right)\right),$$

where Φ is the cumulative distribution function of the Gaussian distribution. The quality of this approximation is dependent on the quality of the surrogate, which in the case of BO is often good near the optimal parameter values, but might be worse further away. However, as we are most interested in estimating the peak of the distribution accurately, this trade‐off is sensible.

There are certain key benefits of computing the posterior, over just performing BO for optimizing the model fit. First, using probability distributions allows combining evidence from multiple sources in a principled way. For example, it is easy to use prior distributions for adding sensible soft constraints to model posterior. In comparison, it is difficult to prevent a pure optimization algorithm from converging to parameter combinations that happen to lead to good predictions, but that are scientifically implausible. Second, the posterior can be used as a prior distribution in a follow‐up study, for example, if further experiments are conducted. In comparison, it is not clear how a plain model fit surface from a previous study should influence the inference process in a follow‐up study. Third, the probability distribution provides intuitive answers to how likely certain parameter values are compared to others, as the ratio of corresponding posterior probabilities directly answers this question. In comparison, a plain model fit surface is only informative of how much the model fit changes between locations in the parameter space. Fourth, samples from the probability distribution can be computed in a principled manner, for example, with Markov chain Monte Carlo (MCMC) methods. This allows one to estimate, for example, the posterior predictive distribution^6^ of the cognitive model. In comparison, there is no straightforward way to perform this with just a plain model fit surface.

## 4 General method: Worked examples

In the following two examples, we evaluate the efficiency and informativeness of four different inference methods. Our goal is to demonstrate the drawbacks and benefits of the methods, as described in the previous section, as well as provide two worked examples on how to utilize the methods in parameter inference of cognitive models.

### 4.1 Inference methods

In addition to grid search and Nelder–Mead optimization, we use the two methods described in the previous section. The first method is BO. The acquisition function used for selecting the parameter values for evaluating the model is a UCB rule for model fit (Srinivas, Krause, Seeger, & Kakade, 2010). When optimizing in parallel batches, we additionally use a local penalization rule for “spreading out” the parameter values within each batch (González, Dai, Hennig, & Lawrence, 2016). Finally, when the maximum number of model evaluations is reached, the parameter values where the surrogate predicts the best expected model fit are selected.^7^

The second additional method is ABC based on the GP model constructed during the BO process. First, the approximate likelihood is estimated using the GP model. Second, the approximate posterior of the model parameters is constructed by multiplying the likelihood with the prior. Finally, the mean of the approximate posterior is estimated using MCMC sampling, and selected as the parameter point estimate. For this reason, this specific method is denoted as ABC PM later on (PM for posterior mean).

### 4.2 Method comparison

The efficiency of the methods was evaluated by measuring how good model fit each method was able to produce as a function of the used computational resources. Model fit quality was measured using predictive error on a separate hold‐out dataset, in a small region around the final parameter estimate.^8^ As the methods were given equal computational hardware, the resource use was measured in elapsed wall‐clock time. Multiple independent replications of the inference processes were conducted with different random seeds to estimate the average performance as well as the variability. Care was taken to implement the methods in a computationally efficient manner, and to manually tune the hyperparameters of each method, so that they each performed as well as one could hope for.

The informativeness of the methods was evaluated by plotting either the predicted model fit or posterior distribution as a graph. As the parameter spaces are high‐dimensional, 2D and 1D slices centered around the optimal parameter values were used for visualization. The overall informativeness of these graphs was evaluated visually.

### 4.3 The example models

As examples we use two popular model families from the cognitive science literature. We give here a quick overview of the models and the rationale for their selection. Further details of the models themselves are provided in the introduction of each worked example.

The first is an ACT‐R model that predicts the cognitive behavior associated with learning how new mathematical operations work. The ACT‐R model family has a long history in cognitive science, but no best practices for parameter inference have been established for this model. We are able to demonstrate that principled posterior inference is possible for this model family, without having to resort to model‐specific methods, which has a potentially high impact to the field. Another reason for choosing this model is that it is a highly complex simulator model. Hence, it would be highly impractical for most practitioners to try to reverse engineer the implementation, for example, in order to derive a purely mathematical representation of the model. Thus, in practice, only general‐purpose inference techniques are applicable for this model.

The second example is a model based on computational rationality, which uses reinforcement learning (RL) for modeling the visual search behavior of a computer user searching through a vertical menu. One compelling reason for selecting this model as an example is the significantly long runtime. While the ACT‐R model takes tens of seconds to execute, the RL optimization required to find the optimal behavioral policy takes multiple hours in comparison. This presents a large challenge for parameter inference, as one can hope to evaluate the model at most with 10 different parameter values per day if using a single CPU. However, we are able to demonstrate that reliable automated inference is still possible even for models of this complexity, thanks to the sample‐efficiency of BO.

### 4.4 Implementation details

For grid search, the implementation was done by authors. For Nelder–Mead, we used the implementation from the open‐source SciPy Python library. For BO and ABC, we used the open‐source ELFI library (Lintusaari et al., 2017), with some additional methods implemented by the authors. Python 3 was used for all implementation, except for the ACT‐R model, which was implemented in Common LISP and acquired from the ACT‐R website. All code implemented by the authors for this paper are available at https://github.com/akangasr/cogsciabc.

The methods were executed on a computing cluster with Intel Xeon 2.7 GHz 6‐core processors. The inference for the ACT‐R model was not parallelized, and the inference was limited to 3 GB of memory. The RL model was parallelized using 20 worker processes, each limited to 6 GB of memory. However, Nelder–Mead was executed in a single process as the method does not parallelize as such. As the same computing environment was used for both experiments, the CPU‐hours reported later are comparable.

## 5 Example 1: ACT‐R

ACT‐R is a cognitive architecture which consists of interconnected modules simulating cognitive and behavioral processes. ACT‐R has a long history, and its modules and module interactions have been extensively tested and validated in both laboratory (Anderson, 2007; Anderson & Betz, 2001; Gunzelmann & Lyon, 2011) and real‐life tasks (Bellamy, John, & Kogan, 2011; Ehret, 2002; John, Prevas, Salvucci, & Koedinger, 2004).

The foundational assumption in all ACT‐R models is that cognition and behavior are a consequence of interaction between the architecture's modules, such as declarative and procedural memory, vision, and manual control. This enables modeling of complex cognitive phenomena, for instance, skill acquisition and multitasking. The module‐based approach also makes it possible to test the model predictions using neuroimaging, under the assumption that processing in different modules activates different regions in the brain.

Each of ACT‐R's modules has a number of parameters which can be freely set by the modeler. Many of the parameters are used to scale time‐based predictions by the model. The associative long‐term memory module, for example, predicts memory retrieval, and its speed and accuracy can be shaped with parameters, such as noise and latency factor. Similarly, the vision module simulates visual search and encoding of objects, and can be governed by parameters which affect the speed of eye movements and encoding.

### 5.1 Traditional parameter inference

The inference of ACT‐R model parameters has not always been documented in detail. It is not uncommon to see casual descriptions of the procedure, such as “control and vary [parameter values] to fit [model] to data” (Servan‐Schreiber & Anderson, 1990, p. 604), “[estimating parameter values] without carefully optimizing our [model] fit” (Anderson, Bothell, Lebiere, & Matessa, 1998, p. 356), or plainly stating that the parameter values were estimated without explication of the procedure (Anderson & Matessa, 1998; Anderson & Reder, 1999; Lovett, Daily, & Reder, 2000). Sometimes, the parameters and their inference are not explicitly mentioned at all (Corbett & Anderson, 1994). The ACT‐R reference implementation (Bothell, 2017) specifies a default, empirically established value for each parameter, and often models retain these values as long as they produce an acceptable model fit (Altmann & Gray, 1998; Lovett et al., 2000).

However, there are also ACT‐R modeling studies with more focus on parameter inference. For example, Anderson, Reder, and Lebiere (1996) investigated the effect of various parameter values on the overall model fit by performing sensitivity analysis, where they manipulated the model parameters one by one and demonstrated how the model fit changes. There is also a recent line of research focused on constructing fully analytic ACT‐R models (Said et al., 2016), which could allow more efficient parameter inference using, for example, gradient‐based methods. Some recent ACT‐R studies have also used neuroimaging data to isolate active mental stages during task completion and used these measurements to infer specific model parameters (Anderson & Fincham, 2014a; Zhang, Walsh, & Anderson, 2016).

### 5.2 Skill acquisition model

Tenison and colleagues (Tenison & Anderson, 2016; Tenison, Fincham, & Anderson, 2016) constructed an ACT‐R model to simulate the distinct phases of skill acquisition. Their skill model is based on the three phases suggested by Fitts and Posner (1967): cognitive, associative, and autonomous, which the authors connect to different modules of the ACT‐R architecture. The authors modeled numerical problem solving, and predicted how humans gradually get better at solving the problem, first by arithmetic computation, then by retrieval of the correct answer, and finally, by automatic manual response to a recognized problem. By modeling transitions between these stages, the authors are able to predict both behavioral and neural responses of human participants. The model had three distinct phases of problem solving: encode the problem, solve the problem, and respond to the problem. The authors measured how long the participants took in each of these stages, given the difficulty of the problem and the cognitive stage of learning, and fitted their model predictions to these observations.

The authors based most of the model parameters on an earlier model, where the authors had fitted eight parameters of the problem‐solving model to human data using an unmentioned inference method (Anderson & Fincham, 2014b). The parameters dictated the behavior of different modules of ACT‐R, such as metacognition processing time or the time it takes for the manual module to type in an answer. This skill phase adopted the eight parameter values from the earlier study, and fit the values of the two new parameters manually (Tenison et al., 2016).

### 5.3 Methods

We set out to infer the values of four selected parameters which we estimated to have the largest effect on model fit. The parameters and their plausible ranges were: retrieval threshold RT ∈ [−4.5, −2.5], retrieval latency factor LF ∈ [0.001, 0.15], base‐level constant activation BLC ∈ [0, 20], and activation noise ANS ∈ [0.001, 0.15]. The ranges were estimated manually, taking into account their plausibility and avoiding regions where the model occasionally crashed during evaluation. The values inferred in the original paper were: RT −2.6, LF 0.1, BLC 2.0, and ANS 0.0.

We quantified the model fit using the root mean square error (RMSE) in the means of the learning stage durations:

$$RMSE:=\sqrt{\frac{1}{9}\sum_{phase\in [1,2,3]}\sum_{stage\in [1,2,3]}{\left(d_{obs,phase,stage}-d_{pred,phase,stage}\right)}^{2}}.$$

As we only had access to the aggregate observation data, we were not able to divide the observations into separate training and test sets. For that reason, the same dataset was used for both training and testing. This unfortunately prevents us from evaluating the extent of overfitting to the training data in this case.

For posterior estimation, we used a prior which was constructed such that values commonly used in ACT‐R models had higher prior probability. These values were estimated based on the extensive library of example models provided along the ACT‐R software library (Bothell, 2017). For RT, we used a uniform prior, as there is large variability in the values, from −1,000 up to 5. For LF, we used a normal distribution with mean 0.2 and standard deviation 0.2, which is based on most values being between 0 and 0.4. For BLC, we used a normal distribution with mean 10 and standard deviation 10. As the only value encountered was 10, we assume that 10 is a reasonable mean and that similarly reasonable values are between 0 and 20. For ANS, we used a normal distribution with mean 0.3 and standard deviation 0.2, which is based on most values being between 0.1 and 0.5.

### 5.4 Results

Given around 6 h of CPU‐time for each method, the results were as follows. With grid search, we obtained parameter values RT −3.0, ANS 0.06, LF 0.07, and BLC 14.3. With Nelder–Mead, we obtained values RT −2.9, ANS 0.03, LF 0.070, and BLC 16.9. The BO‐based values were RT −2.9, ANS 0.001, LF 0.07, and BLC 6.9. The ABC‐based values were RT −2.9, ANS 0.08, LF 0.07, and BLC 12.2. Comparing the results, we observe that all methods tend to agree with the optimal value of RT, which was around −2.9, and LF, which was around 0.07. This is also close to the values or RT −2.6 and LF 0.1 used in the original paper. For ANS and BLC, the methods tend to disagree with the optimal values. This is explained by the fact that the model seems to be much less sensitive to the values of these two parameters, as will become clear in later analysis (Figs. 2–4).

#### 5.4.1 Efficiency

**Figure 1:**
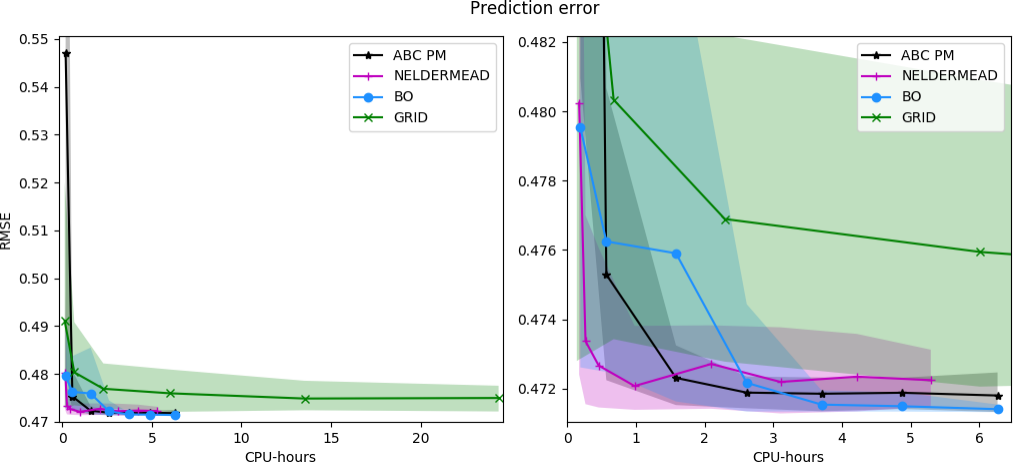
Average model fit and confidence bounds for different point estimation methods as a function of the number of total CPU‐hours used. The shaded region indicates the area between the 5th and 95th percentiles. ABC PM is the ABC posterior mean; BO is Bayesian optimization. Left: Overall behavior. Right: Detail of lower left corner. Each point is estimated using 40 independent experiments.

The efficiency of different inference methods, quantified by the model fits achievable with given computational resources, is visualized in Fig. 1. For all methods, we observe a clear improvement in the model fit as we use more computational resources. However, the rate of improvement depends on the method.

Nelder–Mead is the most efficient in this case, achieving approximate convergence after roughly one CPU‐hour. This efficiency is explained by the fact that the model fit surface is unimodal in this case, which makes local optimization an efficient heuristic, as simply following the surface gradient generally always improves the estimate. However, it can also be seen that Nelder–Mead does not achieve perfect convergence in this case, illustrated by the fact that the confidence bounds do not shrink to zero. This is explained by the fact that the method does not take the slight stochasticity of the model fit into account, and thus may not always converge exactly to the true optimum.

Bayesian optimization and ABC PM are the second most efficient, achieving convergence after roughly three CPU‐hours. The slower convergence speed is explained by the use of global optimization in both cases: the methods need to explore the parameter space sufficiently at the beginning, in order to guarantee that they find the global optimum. BO achieves the best final model fit after around six CPU‐hours. After around four hours of CPU‐time, the result given by BO is essentially better or equally good compared to the estimate of any of the other methods.

Regarding ABC PM, at the beginning, the estimates it makes are very noisy, as the GP surrogate is not yet a good approximation of the model fit surface. This is clearly shown in Fig. 1 left hand side. However, after the GP approximation is sufficient, the model fit starts converging rapidly. The estimate also converges nearly to the optimum solution when measured just by the model fit; this difference is explained by the effect of the prior distribution, which balances the model fit and parameter credibility given prior knowledge. The slight variability in the final estimate is explained by the fact that a finite sample (20k MCMC samples) is used to estimate the posterior mean.

Grid search is the least efficient to converge, and would likely require many orders of magnitude more CPU‐hours to converge to the same quality of parameters as the other methods. Although the method is able to converge relatively quickly at the beginning, the final convergence is very slow and there is significant amount of variation in the results, caused by the variation in the precise locations of the grid cells.

#### 5.4.2 Informativeness

**Figure 2:**
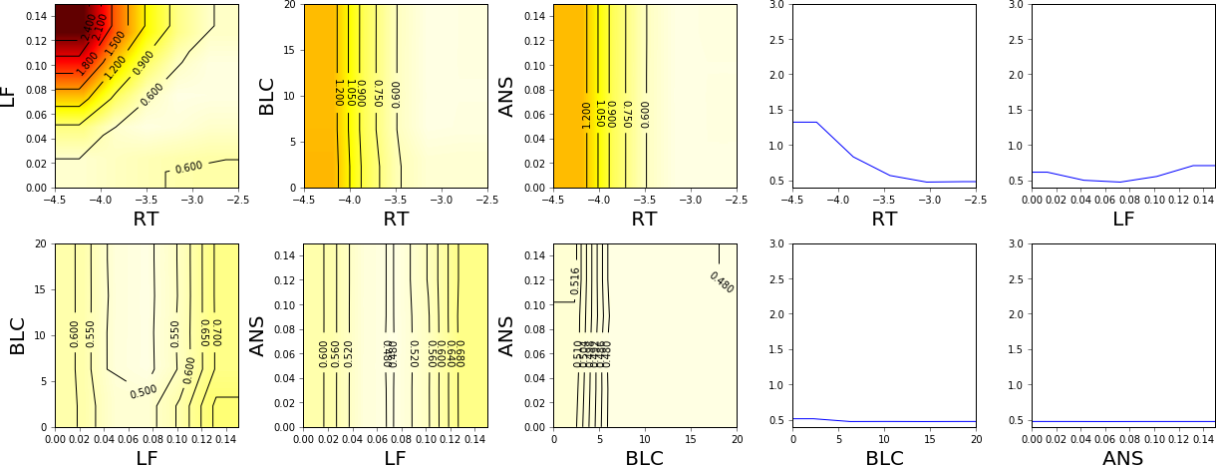
Estimated model fit (2D and 1D slices) near the optimum value (RT −3.0, ANS 0.06, LF 0.07, BLC 14.3) using grid search and 6 CPU‐hours of computation (625 model evaluations). Linear interpolation and constant extrapolation is used between sampled values. The color map is such that black is 3.0 and white is 0.4; lighter shades indicate better model fit. Contours are superimposed for additional clarity.

**Figure 3:**
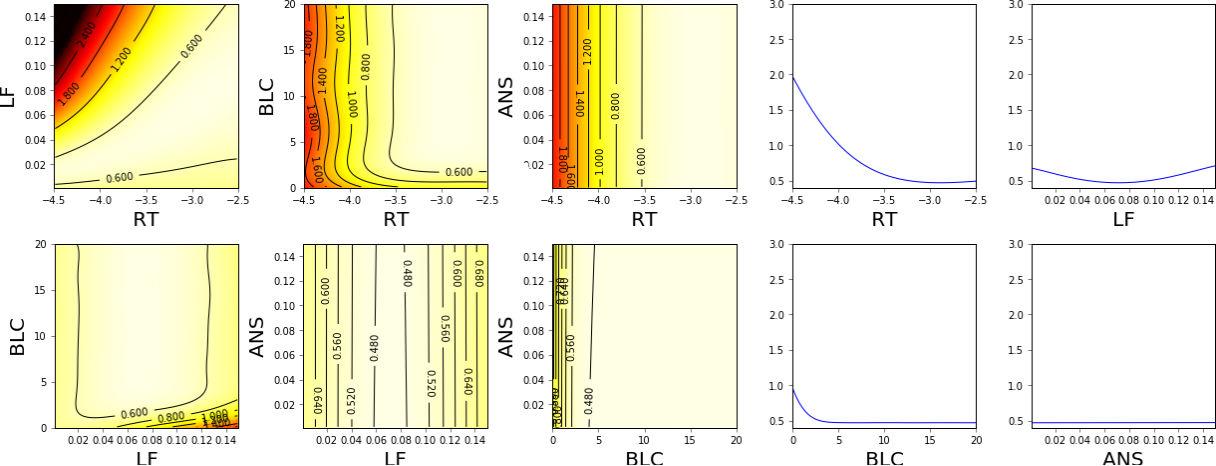
Estimated model fit (2D and 1D slices) near the optimum value (RT −2.9, ANS 0.001, LF 0.07, BLC 6.9) using Bayesian optimization and 5 CPU‐hours of computation (450 model evaluations). The color map is such that black is 3.0 and white is 0.4; lighter shades indicate better model fit. Contours are superimposed for additional clarity.

**Figure 4:**
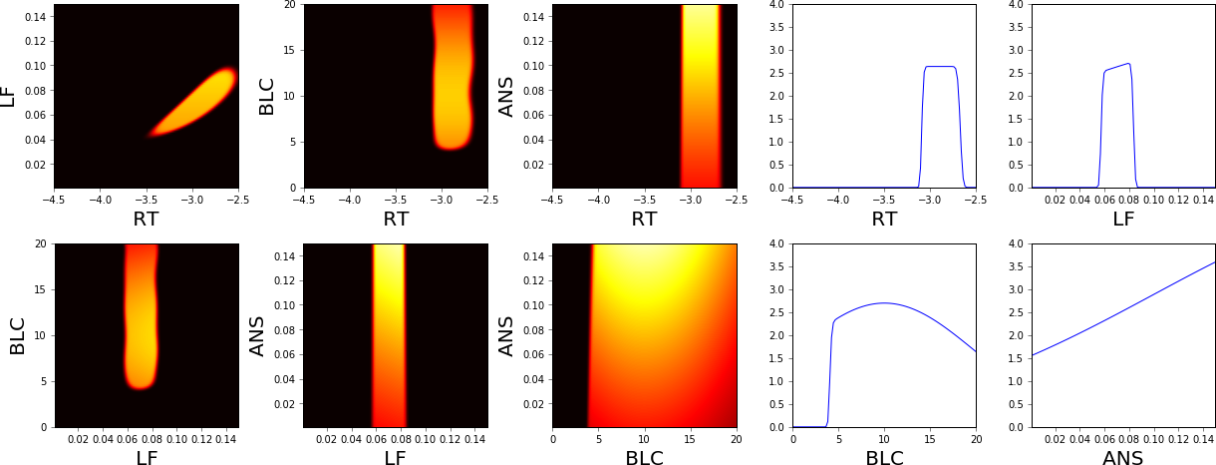
Approximate unnormalized posterior density (2D and 1D slices) near the optimum value (RT −2.9, ANS 0.08, LF 0.07, BLC 12.2) using approximate Bayesian computation and 5 CPU‐hours of computation (450 model evaluations). The ABC threshold ε was 0.48, which is 0.01 above the estimated minimum of the GP surrogate. The color map is such that black is 0.0 and white is 4.0; lighter shades indicate higher posterior probability.

The ability of the different methods to quantify the model fit in different parts of the parameter space is visualized in Figs. 2, 3, 4. As Nelder–Mead only provides a point estimate, no such visualization is possible for it.

Grid search provides the roughest characterization of the model fit across the parameter space, as shown in Fig. 2. As the samples are spread out evenly all across the parameter space, the optimum is not identified very accurately. Furthermore, as no statistical model is used to interpolate the surface and filter out noise, it is not easy to accurately visualize the shape of the surface.

In comparison to the grid visualization, the GP regression model constructed during BO is able to provide a better characterization of the model surface with fewer resources, as shown in Fig. 3. As the sample locations have been optimized by BO, the optimum of the function is estimated with significantly higher precision. Furthermore, the general shape of the model fit function is much easier to interpret from the visualizations, thanks to the statistical interpolation. However, as the optimum is relatively flat in this case, it is still challenging to identify which precise regions of the parameter space are the most likely.

In comparison to the visualized GP model, the ABC posterior, shown in Fig. 4, provides a more intuitive characterization of the likely parameter regions. The overall resolution of the visualization is the same as for the GP model, but the posterior better visualizes the parameter regions that both lead to a good model fit and are probable given our prior understanding of reasonable parameter values. Thus, for example, although different values of the ANS parameter seem to lead to equally good predictions, the posterior shows that based on our prior understanding larger values of ANS should be more likely.

### 5.5 Comparison to manual fitting

**Figure 5:**
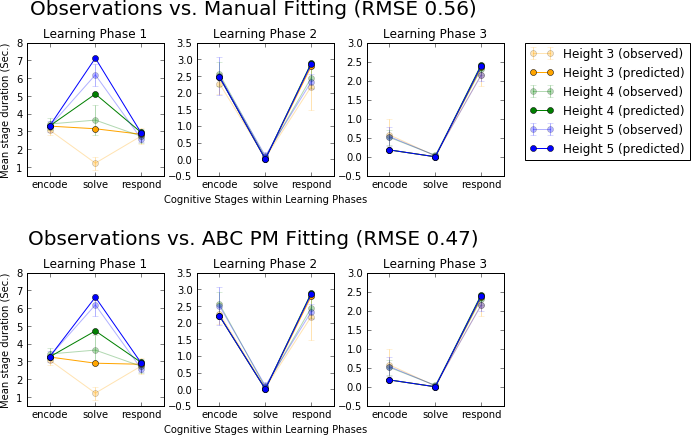
A comparison of observation data and model predictions. ABC PM is ABC posterior mean. Top: Predictions based on manually tuned parameters compared to observation data. Bottom: Predictions based on parameters fit with an automatic inference method (here posterior mean from approximate Bayesian computation) compared to observation data. “Height” refers to the difficulty of the mental task in the experiment that was originally modeled.

We compared the predictions made with an automatic inference method to those manually tuned in the original paper (Tenison et al., 2016). As ground truth, we use the observation data collected in Tenison et al. (2016). The difference to ground truth is visualized in Fig. 5.

In this experiment, the automatic parameter inference leads to 16% reduction in prediction error. This improvement is most notable in learning phase 1. With the manually tuned parameter values, the durations of the solving stages with heights 4 and 5 were over 1 standard deviation away from the observed means, visible in the top left panel of Fig. 5. By automatically tuning the parameter values, these durations are visibly closer to the observation data, as shown in the bottom left panel. For height 3, the predictions are not yet close to the observation data. However, through extensive searching, it is certain that the model is unable to replicate this behavior just by tuning these four parameters within sensible limits. Thus, if one was to continue development of this model, there is now clear evidence of a particular type of behavior that is not reproduced by this model. This would allow one to focus their efforts better, as it is clear that the issue cannot be remedied simply by further tuning of these four parameters.

There is also a slight trade‐off the automatic method makes, visible in the durations of the encoding stages in learning phase 2. However, as there is significant variability in the observation data as well at this point, the predictions are still credible.

## 6 Example 2: Computational rationality

Computational rationality is a framework for cognitive modeling that is based on the idea that cognitive behaviors are generated by *behavioral policies* 9 that are optimally adapted to the processing limits of a cognitive architecture (Gershman, Horvitz, & Tenenbaum, 2015; Griffiths, Lieder, & Goodman, 2015; Howes et al., 2009; Lewis, Howes, & Singh, 2014). In contrast to frameworks such as ACT‐R, which encourage hand‐coding of behavioral policies, computational rationality assumes that these policies emerge from the limitations of the specified cognitive architecture.

As a framework for modeling cognition, computational rationality has been heavily influenced by *rational analysis*, a method for explaining behavior in terms of utility (Anderson, 1991; Chater & Oaksford, 1999; Oaksford & Chater, 1994), an idea used, for example, in information foraging theory and economic models of search (Azzopardi, 2014; Pirolli & Card, 1999). Computational rational agents have been used to model a number of phenomena in HCI (Payne & Howes, 2013). Applications relevant to this paper include menu interaction (Chen et al., 2015), visual search (Hayhoe & Ballard, 2014; Myers, Lewis, & Howes, 2013; Nunez‐Varela & Wyatt, 2013; Tseng & Howes, 2015), and decision‐making (Chen, Starke, Baber, & Howes, 2017).

A key problem in computational rationality is finding the optimal behavioral policy. One popular approach is to write the optimization problem as a Markov decision problem (MDP) and then use a suitable reinforcement learning algorithm for solving the optimal policy (Sutton & Barto, 1998). The definition of an MPD consists of a set of cognitive states *S*, a set of cognitive actions *A*, the transition dynamics between states based on actions *s*_*t*_ **= *T* (*s*_*t−*1_, *a*_*t−*1_), a reward function *R*(*s*_*t*_) and a temporal discount factor for rewards γ. The behavioral policy is then denoted as *a*_*t*_ **= *π*(*s*_*t*_, ϕ), where ϕ are the parameters solved by the reinforcement learning algorithm to maximize the expected sum of discounted rewards:

(1) $$E\left[\sum_{t}R\left(s_{t}\right)\gamma^{t}\right].$$

The resulting policy—and thus the predicted behavior—naturally depends on the parameters of the MDP, such as the magnitudes of rewards and the stochasticity of state transitions. The MDP is often designed in such a way that these parameters correspond to interesting psychometric quantities, such as the level of motivation or alertness of the subject.

An interesting property of computational rationality, that is, pertinent to the current paper, is that it effectively reduces the number of free parameters in the model. Although in other modeling frameworks the parameters of the behavioral policy can be adjusted freely, computational rationality demands that these parameter values are derived through optimization, given the limits imposed by the cognitive architecture. In models based on computational rationality, the free parameters generally relate to these cognitive limits only. Thus, the number of free parameters that remain to be inferred is often much smaller compared to the number of parameters needed to fully define the behavioral policy.

### 6.1 Traditional parameter inference

While the parameters of the behavioral policy are derived through optimization and are, therefore, not fitted to data, the parameters that define the limitations of the cognitive architecture remain to be inferred. However, a key challenge with this inference process is the fact that evaluating the model fit using new parameter values takes significant time, as the parameters of the behavioral policy need to be solved before predictions can be made. Likely for this reason, the majority of parameters in existing models have been set manually. For example, both Lewis et al. (2014) and Acharya, Chen, Myers, Lewis, and Howes (2017) used grid search for inferring the value of only a single model parameter. It is also not uncommon for the authors to have chosen all of the parameters manually (Chen et al., 2015, 2017).

### 6.2 Menu search model

The model used in this example was introduced recently by Chen et al. (2015) and later extended by Kangasrääsiö et al. (2017). This model predicts the visual search behavior (how eyes fixate and move) and task completion times of a person searching for an item from a computer drop‐down menu. We use here a slightly simplified version of the model, in order to somewhat reduce the time required for solving the optimal policy. This allowed us to run studies with more model evaluations for better demonstrating the convergence properties of the algorithms.

The model structure is as follows. The model contains a menu composed of eight items. The user has a target item in mind, which is either present in (90% of the cases) or absent from (10% of the cases) the menu. The agent has multiple possible actions at each step. She can either fixate on any of the eight items or declare that the item is not present in the menu (i.e., to quit). If the agent fixates on the target item, it is automatically selected. (In the original model, the agent had to manually choose to select the item, which now happens automatically as it is clearly the optimal option at that point.) Fixating on a non‐target item reveals its semantic relevance to the agent, as well as with some probability the length of the item and the semantic relevances and lengths of nearby items as well. Fixating on the target item or quitting ends the episode. The cognitive state *s* consists of the semantic relevances and lengths of the observed menu items. (In the original model, the state also included the location of the previous fixation, but this was determined to have little effect on the policy and was thus left out.) The agent receives a reward after each action. If the agent found the target item, or quit when the target was absent from the menu, a large reward is given. If the agent quits when the target is present, an equally large penalty is given. Otherwise, the agent receives a small penalty, which is equal to the time spent for the action (sum of the durations of the saccade and the fixation).

The optimal behavioral policy is learned using a RL algorithm known as Q‐learning (Watkins & Dayan, 1992). Execution of the Q‐learning algorithm is the main reason for the long runtime of the model. After choosing values for the free model parameters, it takes roughly two hours to estimate a policy which is close to optimal.

In the original paper by Chen et al. (2015), the values of the parameters were set manually to values obtained from earlier literature. For example, the parameters that determine the duration of saccades were set based on a study by Baloh, Sills, Kumley, and Honrubia (1975), and the duration of eye fixations was set based on a study by Brumby, Cox, Chung, and Fernandes (2014). The sensitivity of the model predictions to variation in parameter values was not reported.

Later Kangasrääsiö et al. (2017) used BO and ABC for estimating the maximum of the posterior distribution (MAP estimate) of the parameter values, which improved the model fit. The inference used a dataset collected by Bailly, Oulasvirta, Brumby, and Howes (2014). Our study extends their analysis in multiple ways. First, the full posterior distribution of the model is estimated, instead of just the maximum of the posterior. This provides a rigorous characterization of the remaining uncertainty in the parameter values, which was not discussed in the earlier study. Second, the mean of the posterior is estimated, which is often a more robust point estimate compared to the maximum. Third, the efficiency of the method is rigorously compared to alternative methods, which was not done previously.

### 6.3 Methods

We focus on inferring the values for three model parameters: the duration of fixations *f*_*dur*_ ∈ [0 ms, 500 ms], the duration of the selection of the target item *d*_*sel*_ ∈ [0 s, 1 s], and the probability of recalling the menu layout from memory *p*_*rec*_ ∈ [0, 1]. These parameters were selected as they were judged to have the largest effect to the predicted behavior. Further, it would be very challenging to estimate the selection delay or recall probability based only on earlier literature, as they may be largely affected by the precise setup used to collect the data.

The original model by Chen et al. (2015) had set *f*_*dur*_ manually to 400 ms, and did not yet include the model features related to *d*_*sel*_ or *p*_*rec*_. The corresponding MAP parameter estimates inferred by Kangasrääsiö et al. (2017) were *f*_*dur*_ 280 ms, *d*_*sel*_ 290 ms, and *p*_*rec*_ 0.69. In addition to these parameters, the probability of observing the semantic similarity of neighboring items with peripheral vision was inferred to be 0.93. In this study, we use this constant value for this parameter as we assumed it would have the smallest effect on the performance, and as the model is expensive to evaluate, using fewer parameters allowed us to run more a extensive comparison study.

We quantify the model fit by the natural (base *e*) logarithm of the following error *E*, which is based on the task completion time means μ and standard deviations σ, when the target was present (*pre*) and absent (*abs*) from the menu:

$$E:=\sum_{c\in \{pre,abs\}}\left({\left(\mu_{c,abs}-\mu_{c,sim}\right)}^{2}+\left|\sigma_{c,abs}-\sigma_{c,sim}\right|\right).$$

Both μ and σ are in units of 1 ms.

The users in the dataset collected by Bailly et al. (2014) were divided randomly into two groups. The data from the first group (user IDs 4, 18, 19, 21, 23, 37, 38, 39, 40, and 42) were used only for parameter inference, while the data from the second group (user IDs 5, 6, 7, 8, 20, 22, 24, 36, and 41) were used only for estimating the prediction error.

For posterior estimation, we used a prior very similar to that used by Kangasrääsiö et al. (2017). For *f*_*dur*_, the prior is a normal distribution with mean 300 ms and standard deviation 100 ms, as we believe plausible fixation durations are between 200 ms and 400 ms. For *d*_*sel*_, the prior is a normal distribution with mean 300 ms and standard deviation 300 ms, as we believe plausible fixation durations are between 0 ms and 600 ms. For *p*_*rec*_, the prior is a beta distribution with α* = *3.0 and β* = *1.35, which roughly corresponds to a normal distribution with mean 0.69 and standard deviation 0.2.

As each model evaluation took multiple hours, we used 20 parallel computers to reduce the used wall‐clock time. Although Nelder–Mead is not a parallelizable method, it is possible to run multiple instances of the optimization algorithm in parallel, and select the best overall result. In our experiment, we report both the performance of non‐parallelized Nelder–Mead, as well as a simulation of the parallelized performance, which is computed using the results from the non‐parallelized experiments. In the simulations, for each datapoint, we sampled five experiments without replacement from the corresponding 10 independent experiments and selected the parameter location with smallest error on training data. Five samples were used as this resulted in comparable amount of total CPU‐hours being spent as with the other parallelized methods for the RL model, without sacrificing too much of the mutual independence of the simulated results.

### 6.4 Results

Given around 700 h of CPU‐time for each method, the results were as follows. With grid search, we obtained values *f*_*dur*_ 260 ms, *d*_*sel*_ 150 ms, and *p*_*rec*_ 0.61. The Nelder–Mead optimized values were *f*_*dur*_ 220 ms, *d*_*sel*_ 290 ms, and *p*_*rec*_ 0.51. With BO, the values were *f*_*dur*_ 150 ms, *d*_*sel*_ 430 ms, and *p*_*rec*_ 0.22; and for ABC, they were *f*_*dur*_ 220 ms, *d*_*sel*_ 290 ms, and *p*_*rec*_ 0.58. Comparing the results, we observe that here is more overall disagreement regarding the optimal parameters between the methods than in Example 1. One contributing factor is likely the fact that none of the methods has properly converged within the available computation time caused by the expensive model evaluations. This means that there is likely considerable remaining uncertainty about the location of the best possible parameter values with all of these methods. Out of the compared methods, ABC has the most visually intuitive quantification of this uncertainty (Fig. 9).

#### 6.4.1 Efficiency

**Figure 6:**
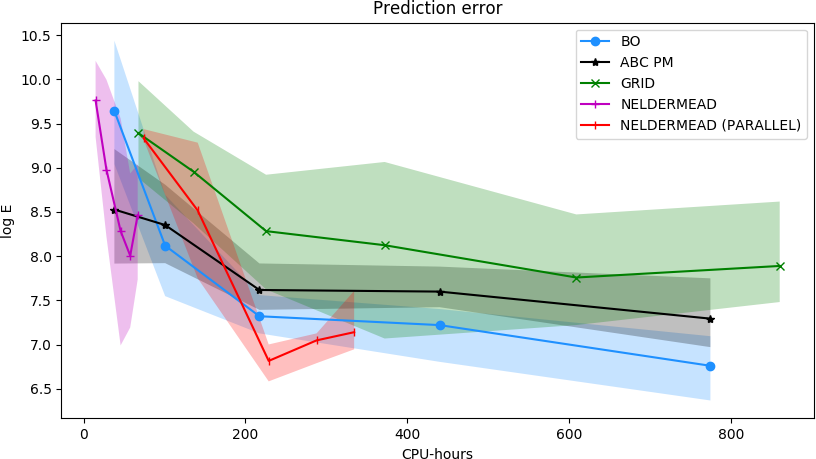
Average model fit and confidence bounds for different point estimation methods as a function of the number of total CPU‐hours used. The shaded region indicates the area between the 25th and 75th percentiles. ABC PM is the ABC posterior mean; BO is Bayesian optimization. Each point is estimated using 10 independent experiments, except for parallel Nelder–Mead, which is simulated using the non‐parallel experiments.

The efficiency of different inference methods, quantified by the model fits achievable with given computational resources, is visualized in Fig. 6. Nelder–Mead is the most efficient to converge, although it is also the most susceptible for over‐fitting the parameters to the training data. This is demonstrated by the fact that prediction error starts increasing after a certain amount of optimization has been performed. This behavior can be expected, as the model evaluations are very stochastic while the method assumes non‐stochastic evaluations. This leads to the method making overly optimistic assumptions about the model fit, based on chance occurrences where the model fit happened to be lower than the average fit achieved with those parameter values. This over‐fitting could be alleviated by analyzing the prediction error on test data, and stopping the optimization when the prediction error on test data starts increasing.

Bayesian optimization is also efficient to converge, being slightly faster at start compared to parallelized Nelder–Mead, but slower to fine‐tune the parameter estimates later on. However, as the method takes the stochasticity of the model evaluations in account, it is also more robust against stochasticity‐related over‐fitting. ABC PM behaves roughly in a similar fashion as BO. In this case, as the prior is more restrictive, the method is able to achieve good model fit even at the beginning of the inference process. As the method optimizes a balance between model fit and credibility of parameter values, the final model fit is higher compared to BO or Nelder–Mead. Grid search is the least efficient to converge, and would likely require many orders of magnitude more CPU‐hours to converge to the same quality of parameters as the other methods.

#### 6.4.2 Informativeness

**Figure 7:**
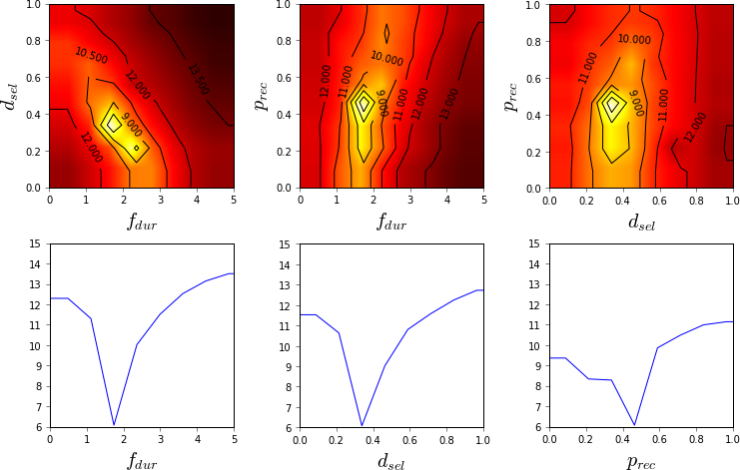
Estimated model fit (2D and 1D slices) near the optimum value (*f*_*dur*_ 260 ms, *d*_*sel*_ 150 ms, *p*_*rec*_ 0.61) using grid search and 660 CPU‐hours of computation (512 model evaluations, 33 h of wall‐clock time). Linear interpolation and constant extrapolation are used between sampled values. The color map is such that black is 15 and white is 6; lighter shades indicate better model fit. Contours are superimposed for additional clarity.

**Figure 8:**
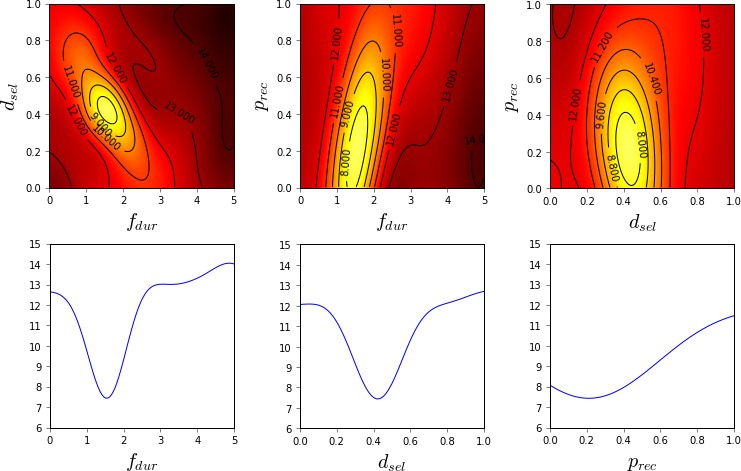
Estimated model fit (2D and 1D slices) near the optimum value (*f*_*dur*_ 150 ms, *d*_*sel*_ 430 ms, *p*_*rec*_ 0.22) using Bayesian optimization and 560 CPU‐hours of computation (420 model evaluations, 28 h of wall‐clock time). The color map is such that black is 15 and white is 6; lighter shades indicate better model fit. Contours are superimposed for additional clarity.

**Figure 9:**
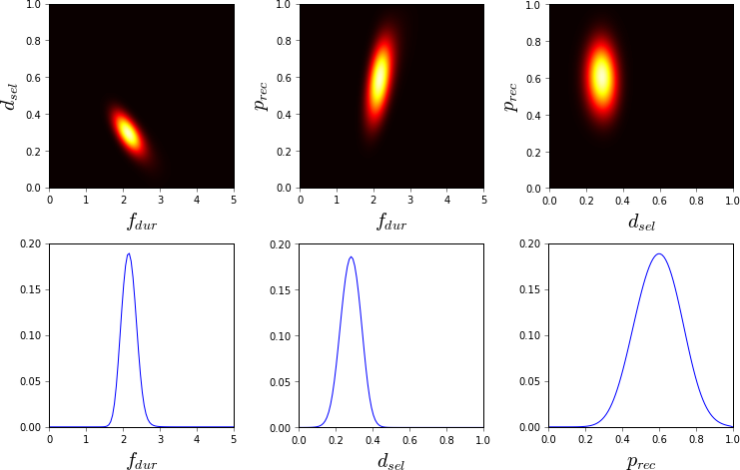
Approximate unnormalized posterior density (2D and 1D slices) near the optimum value (*f*_*dur*_ 220 ms, *d*_*sel*_ 290 ms, *p*_*rec*_ 0.58) using approximate Bayesian computation and 560 CPU‐hours of computation (420 model evaluations, 28 h of wall‐clock time). The ABC threshold ε was 7.45, which is 0.01 above the estimated minimum of the Gaussian process surrogate. The color map is such that black is 0.0 and white is 0.2; lighter shades indicate higher posterior probability.

The ability of the methods to quantify model fit in different parts of the parameter space is visualized in Figs. 7, 8, 9. As Nelder–Mead only provides a point estimate, no such visualization is possible with it.

Similarly as with the ACT‐R model, grid search provides the roughest characterization of the model fit across the parameter space, as shown in Fig. 7, while the GP regression model from BO is able to provide a much better characterization of the model surface using fewer simulations from the model, as shown in Fig. 8. And again, the ABC posterior, shown in Fig. 9, provides an intuitive characterization of the likely parameter regions.

In this example, we notice a clear difference between the optima of the BO model fit surface and the ABC posterior. This is explained by the more restrictive prior distribution used. In this case, the feature that is most restricted by the prior is the fixation duration. While the minimum of the model fit surface predicts a mean fixation duration of 150 ms, this is a low value compared to existing knowledge about fixation durations, which place the mean around 225–400 ms (Rayner, 1998). As this information is encoded in the prior, the ABC posterior automatically makes a principled trade‐off to balance the plausibility of the parameter values with the accuracy of the predictions. In this case, this leads to a slight increase in the fixation duration (from 150 ms to 220 ms), a reduction in selection delay (from 430 ms to 290 ms), and an increase in the probability of recalling the menu (from 22% to 58%).

#### 6.4.3 Posteriors of individual users

With this model, we also inferred the approximate posteriors of individual subjects from the study by Bailly et al. (2014). We omitted users that had a low number of observations from either menu condition. For the selected users, the lowest number of observations from the “target present” condition was 175 and for the “target absent” condition 21 observations. We were left with seven subjects (subject IDs 5, 18, 19, 24, 37, 39, and 40). We repeated the above inference procedure for the data collected of each of these subjects individually, with the restriction of 400 model evaluations (in batches of 20).

**Figure 10:**
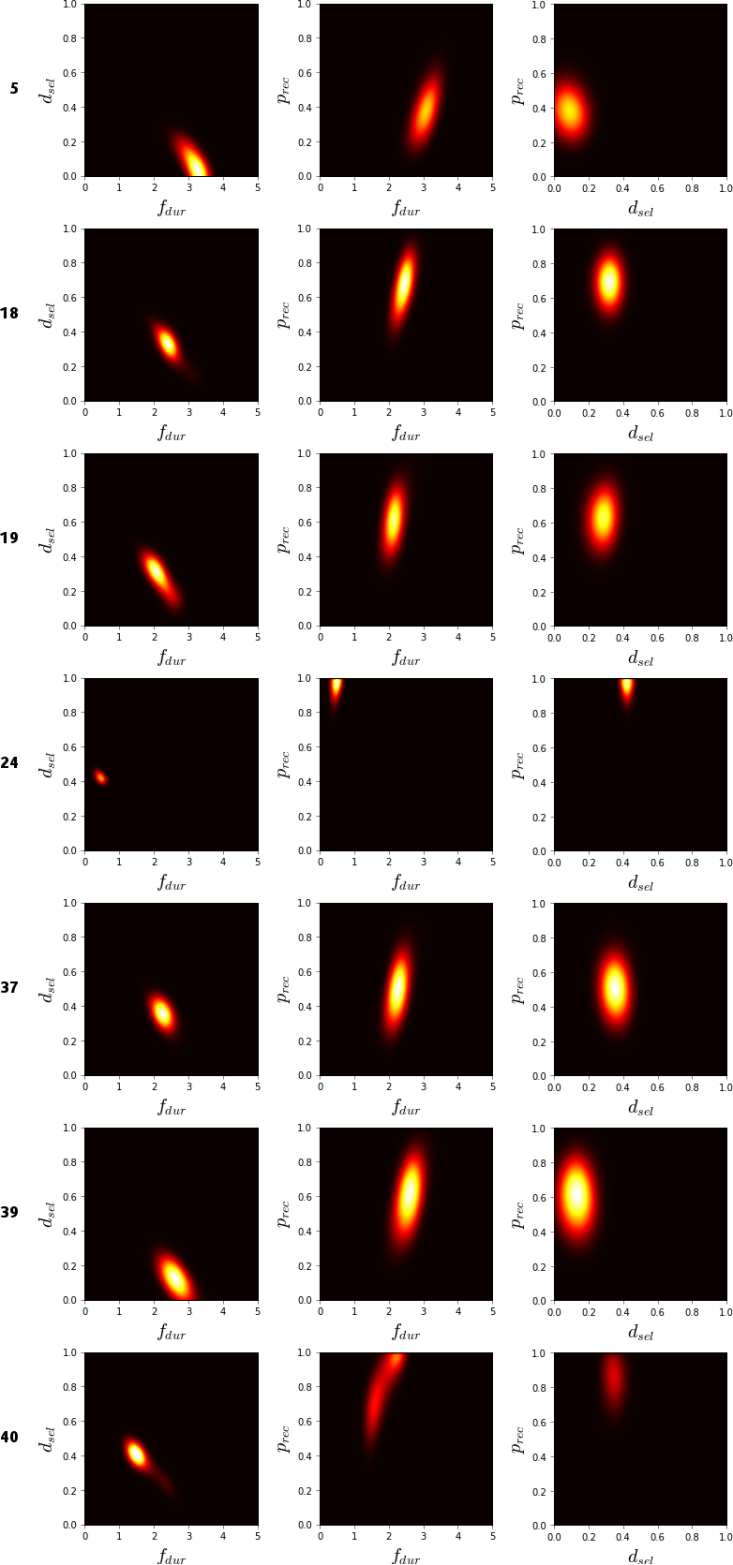
Approximate unnormalized posterior densities (2D slices) near the individual optimum values. One posterior is shown per row, with subject ID on the left. The color map is such that black is 0.0 and white is maximum for each function; lighter shades indicate higher posterior probability.

The estimated posterior distributions are visualized in Fig. 10. We observe that most posteriors are similar in nature as was the population level posterior estimated before, shown in Fig. 9. Especially, subjects 18, 19, and 37 have posteriors that are very similar to the population mean, indicating that a model fit with population level data would be a good approximation for these individuals. However, we also observe clear individual variation in the posteriors, which indicates that the model offers different explanations for the behavior of each individual subject. For example, subjects 5 and 39 had a relatively low selection delay, but in contrast, a slightly longer fixation duration compared to other subjects. We are also able to identify anomalous subjects, such as subject 24, for whom a very long selection delay was inferred, and on the other hand a very short fixation duration, and subject 40, for whom the posterior appears to be more complex and have a heavy tail, which places the posterior mean further away from the posterior maximum. By examining the behavior of such anomalous users more carefully, it would be possible to either spot oddities in data collection procedures, identify completely new types of user strategies, or point out behaviors that the model is unable to reproduce.

## 7 Discussion

Computational cognitive models generally seek to explain aspects of human cognition. However, arguably, the quality of these explanations has too often been undermined by misgivings regarding the parameter inference process. Many models have been published with parameter values that are difficult to justify; sometimes because the inference method is ad hoc or not reported, sometimes because no alternative parameter values have even been considered. Possible reasons for this include the inherent complexity and long run‐times of modern computational models, which prevent the use of certain standard inference methods, such as gradient descent, and make other methods, such as grid search, computationally infeasible. Another reason might be that while a lot of progress has happened in computational statistics in terms of readily applicable inference methods, these have not yet been fully discovered in mainstream cognitive scientific computational modeling. To remedy this situation, we reported an exploration of how principled and rigorous parameter inference can be performed for some of the most complex computational cognitive model families. We compared two relatively recent methods, BO and ABC to two traditional methods, grid search and Nelder–Mead optimization.

The worked examples presented in this paper illustrate the benefits of both BO and ABC. One major benefit is that estimates of parameter values, along with their uncertainty, can be inferred efficiently for various types of computational cognitive models. We found this for both for an ACT‐R model and a reinforcement learning‐based computationally rational model. We note that neither of these models has a tractable likelihood function, which renders many traditional inference methods, such as gradient descent, infeasible. However, we observe that efficient general‐purpose inference techniques such as BO and ABC are able to solve the parameter inference problem reliably regardless of this limitation.

Our results also confirm the common observation that automated parameter estimation methods improve model fit over manual fitting. We demonstrated significant improvements in model fit over a result obtained with manual fitting of the parameters of an ACT‐R model in a previous paper. We also note that the use of automated methods in general insists on explication of model fit functions, search methods, and search spaces, subjecting them to transparency and opening up the potential for scrutiny by the community.

Based on comparisons of different inference methods in two worked examples, we observe that there appears to be a fundamental trade‐off between efficiency and informativeness of different inference methods. In order to get estimates quickly, it is not possible to estimate the model fit over the entire parameter space at the same time, and vice versa. We observed that different methods are able to make this trade‐off more efficiently than others; for example, BO was in both cases more efficient and more informative compared to grid search.

We give two suggestions for selecting inference methods, depending on the situation. While they are based on the two case studies presented herein, they are in line with previous applications.

- If the goal is to obtain reasonable parameter estimates quickly and there is no reason to believe that the parameter space has multiple optima, local optimization‐based methods such as Nelder–Mead are sufficient. Local optimization appears to be able to give reliably good parameter estimates in these cases, although stochastic model evaluations may take the estimates off‐track, and no estimate of parameter uncertainty is given. Example use cases include initial hypothesis testing and early model development.
- If the goal is to obtain robust parameter estimates accompanied by estimates of the sensitivity of parameters, we suggest that methods based on efficient global optimization should be used. These methods are able to estimate model fit across the entire parameter space, while also facilitating the search of optimal values. Based on our experiments, we observed that ABC is an efficient and informative inference method. The method also allows prior information of reasonable parameter values to be taken into account in a principled way.

There are multiple reasons why it is important to estimate the posterior distribution of parameters over just the point estimates. The posterior probability distribution over the parameter values is a rigorously defined quantitative measure of our knowledge about the true parameter values. It grants proper, quantified estimates of uncertainties associated with parameter values, which is inherently valuable for understanding the models and the behavior that they describe. The posterior distribution is also a valuable diagnostic tool in modeling. For example, the shape of the posterior can be informative of insignificant or poorly identified parameters. If the posterior of a certain parameter is flat, this means that either this parameter has no effect on the model predictions, or that there are insufficient observation data to infer the value of this parameter. The posterior shape can also inform alternative explanations; if there are multiple modes in the posterior distribution, this indicates the existence of multiple alternative explanations to the data. Finally, accounting for the stochasticity of the predictions allows comparing their variance to the data using specific parameter values. A complete check of model fit also takes the uncertainty from model fitting into account when estimating the quality of the predictions.

Therefore, parameter inference plays a more decisive role in scientific modeling than just the determination of reasonable parameter values. It is via parameter inference that theories and models gain contact with reality, quantified by the observation data. In general, a good explanatory model should be such that given observation data, the model explicitly informs us about what we can and cannot tell about the unobserved quantities of the cognitive system based on the data, and how reliable these estimates are. However, being able to access such information is only possible through principled parameter inference methods, such as those based on Bayesian statistics. While various point estimation methods, like Nelder–Mead, may quickly find parameters that allow replicating the observation data, probabilistic methods that consider the parameter space as a whole, like ABC, allow answering the above questions more robustly. Such an inspection of the parameter space could also be useful for assessing how the model constrains possible predictions (Roberts & Pashler, 2000).

In conclusion, modern solutions to the parameter inference problem have the potential to transform the rigor, transparency, and efficiency of computational cognitive modeling. Recent statistical inference methods, such as BO and ABC, can be used for inferring the parameter values for some of the most complex simulation models developed in the field of cognitive science. As argued here, these methods have important advantages compared to the traditional methods. In the future, we hope that these inference methods make it feasible for researchers in the field to work on even more ambitious computational cognitive models.

## Notes

1 Assuming comparable hardware between methods.

2 The term *P*(*D*_*obs*_) is the marginal likelihood of observing *D*_*obs*_ considering all possible parameter values.

3 The notation *x* ∼ *P*(*x*) indicates values of *x* are drawn independent and identically distributed from probability distribution *P*(*x*).

4 The gradient is the direction in the parameter space which provides largest improvement in the model fit, assuming small changes in parameter values.

5 Nelder–Mead further belongs to the subclass of “direct search” methods (Kolda, Lewis, & Torczon, 2003), meaning that it only requires ordinal comparisons between function values.

6 The posterior predictive distribution estimates the distribution of plausible behaviors, taking into account the uncertainty in the model parameters.

7 Restricted to locations in the parameter space where we actually have evaluated the model during inference, to reduce errors from extra‐ and interpolation.

8 Ten estimation locations were drawn from a uniform distribution that was centered at the point estimate and had a width of 2% of the respective bound width. For example, if the point estimate was 0.5 and the bounds were [0, 1], we would sample 10 locations from Uniform(0.49, 0.51).

9 By behavioral policy, we mean a decision rule which chooses what cognitive action is to be executed in each possible cognitive state.

## References

[cogs12738-bib-0001] Abel, L. A. , Troost, B. T. , & Dell'Osso, L. F. (1983). The effects of age on normal saccadic characteristics and their variability. Vision Research, 23(1), 33–37. 6868379 10.1016/0042-6989(83)90038-x

[cogs12738-bib-0002] Acharya, A. , Chen, X. , Myers, C. W. , Lewis, R. L. , & Howes, A. (2017). Human visual search as a deep reinforcement learning solution to a POMDP In Proceedings of the 39th Annual Conference of the Cognitive Science Society.

[cogs12738-bib-0003] Altmann, E. M. , & Gray, W. D. (1998). Pervasive episodic memory: Evidence from a control‐of‐attention paradigm In Proceedings of the Twentieth Annual Conference of the Cognitive Science Society (pp. 42–47).

[cogs12738-bib-0004] Altmann, E. M. , & Trafton, J. G. (2002). Memory for goals: An activation‐based model. Cognitive Science, 26(1), 39–83.

[cogs12738-bib-0005] Anderson, J. R. (1991). Is human cognition adaptive? Behavioral and Brain Sciences, 14(3), 471–485. 10.1017/S0140525X00070801.

[cogs12738-bib-0006] Anderson, J. R. (2007). How can the human mind occur in the physical universe? New York, NY: Oxford University Press.

[cogs12738-bib-0007] Anderson, J. R. , & Betz, J. (2001). A hybrid model of categorization. Psychonomic Bulletin & Review, 8(4), 629–647. 11848582 10.3758/bf03196200

[cogs12738-bib-0008] Anderson, J. R. , Bothell, D. , Byrne, M. D. , Douglass, S. , Lebiere, C. , & Qin, Y. (2004). An integrated theory of the mind. Psychological Review, 111(4), 1036. 15482072 10.1037/0033-295X.111.4.1036

[cogs12738-bib-0009] Anderson, J. R. , Bothell, D. , Lebiere, C. , & Matessa, M. (1998). An integrated theory of list memory. Journal of Memory and Language, 38(4), 341–380.

[cogs12738-bib-0010] Anderson, J. R. , & Fincham, J. M. (2014a). Discovering the sequential structure of thought. Cognitive Science, 38(2), 322–352. 23941168 10.1111/cogs.12068

[cogs12738-bib-0011] Anderson, J. R. , & Fincham, J. M. (2014b). Extending problem‐solving procedures through reflection. Cognitive Psychology, 74, 1–34. 25063939 10.1016/j.cogpsych.2014.06.002

[cogs12738-bib-0012] Anderson, J. R. , & Matessa, M. (1998). The rational analysis of categorization and the ACT‐R architecture In Oaksford M. & Chater N. (Eds.), Rational models of cognition (pp. 197–217).

[cogs12738-bib-0013] Anderson, J. R. , & Reder, L. M. (1999). The fan effect: New results and new theories. Journal of Experimental Psychology: General, 128(2), 186–197.

[cogs12738-bib-0014] Anderson, J. R. , Reder, L. M. , & Lebiere, C. (1996). Working memory: Activation limitations on retrieval. Cognitive Psychology, 30(3), 221–256. 8660785 10.1006/cogp.1996.0007

[cogs12738-bib-0015] Azzopardi, L. (2014). Modelling interaction with economic models of search In Proceedings of the 37th international ACM SIGIR conference on research & development in information retrieval (pp. 3–12). ACM 10.1145/2600428.2609574

[cogs12738-bib-0016] Bailly, G. , Oulasvirta, A. , Brumby, D. P. , & Howes, A. (2014). Model of visual search and selection time in linear menus In Proceedings of the 32nd annual ACM conference on human factors in computing systems (pp. 3865–3874). ACM 10.1145/2556288.2557093

[cogs12738-bib-0017] Baloh, R. W. , Sills, A. W. , Kumley, W. E. , & Honrubia, V. (1975). Quantitative measurement of saccade amplitude, duration, and velocity. Neurology, 25(11), 1065 10.1212/WNL.25.11.1065. 1237825

[cogs12738-bib-0018] Bellamy, R. , John, B. , & Kogan, S. (2011). Deploying Cogtool: Integrating quantitative usability assessment into real‐world software development In Proceedings of the 33rd international conference on software engineering (pp. 691–700). ACM.

[cogs12738-bib-0019] Blouw, P. , Solodkin, E. , Thagard, P. , & Eliasmith, C. (2016). Concepts as semantic pointers: A framework and computational model. Cognitive Science, 40(5), 1128–1162. 26235459 10.1111/cogs.12265

[cogs12738-bib-0020] Bogacz, R. , Brown, E. , Moehlis, J. , Holmes, P. , & Cohen, J. D. (2006). The physics of optimal decision making: A formal analysis of models of performance in two‐alternative forced‐choice tasks. Psychological Review, 113(4), 700. 17014301 10.1037/0033-295X.113.4.700

[cogs12738-bib-0021] Bothell, D. (2017). Act‐r 7 reference manual. Retrieved from http://act-r.psy.cmu.edu/actr7/reference-manual.pdf

[cogs12738-bib-0022] Botvinick, M. M. , Braver, T. S. , Barch, D. M. , Carter, C. S. , & Cohen, J. D. (2001). Conflict monitoring and cognitive control. Psychological Review, 108(3), 624. 11488380 10.1037/0033-295x.108.3.624

[cogs12738-bib-0023] Brochu, E. , Cora, V. M. , & De Freitas, N. (2010). A tutorial on Bayesian optimization of expensive cost functions, with application to active user modeling and hierarchical reinforcement learning. ArXiv Preprint *, arXiv:1012.2599*.

[cogs12738-bib-0024] Brumby, D. P. , Cox, A. L. , Chung, J. , & Fernandes, B. (2014). How does knowing what you are looking for change visual search behavior? In Proceedings of the SIGCHI conference on human factors in computing systems (pp. 3895–3898). . 10.1145/2556288.2557064

[cogs12738-bib-0025] Burling, J. M. , & Yoshida, H. (2017). Highlighting in early childhood: Learning biases through attentional shifting. Cognitive Science, 41(S1), 96–119. 27634614 10.1111/cogs.12408 PMC5316359

[cogs12738-bib-0026] Busemeyer, J. R. , & Townsend, J. T. (1993). Decision field theory: A dynamic‐cognitive approach to decision making in an uncertain environment. Psychological Review, 100(3), 432. 8356185 10.1037/0033-295x.100.3.432

[cogs12738-bib-0027] Chater, N. , & Oaksford, M. (1999). Ten years of the rational analysis of cognition. Trends in Cognitive Sciences, 3(2), 57–65. 10.1016/S1364-6613(98)01273-X. 10234228

[cogs12738-bib-0028] Chen, X. , Bailly, G. , Brumby, D. P. , Oulasvirta, A. , & Howes, A. (2015). The emergence of interactive behavior: A model of rational menu search In Proceedings of the 33rd annual ACM conference on human factors in computing systems (pp. 4217–4226). ACM 10.1145/2702123.2702483

[cogs12738-bib-0029] Chen, X. , Starke, S. D. , Baber, C. , & Howes, A. (2017). A cognitive model of how people make decisions through interaction with visual displays In Proceedings of the 2017 CHI conference on human factors in computing systems (pp. 1205–1216). ACM.

[cogs12738-bib-0030] Cooper, R. P. (2002). Model ling high‐level cognitive processes. Lawrence Erlbaum Associates.

[cogs12738-bib-0031] Corbett, A. T. , & Anderson, J. R. (1994). Knowledge tracing: Modeling the acquisition of procedural knowledge. User Modeling and User‐Adapted Interaction, 4(4), 253–278.

[cogs12738-bib-0032] Csilléry, K. , Blum, M. G. , Gaggiotti, O. E. , & François, O. (2010). Approximate Bayesian computation (ABC) in practice. Trends in Ecology & Evolution, 25(7), 410–418. 10.1016/j.tree.2010.04.001. 20488578

[cogs12738-bib-0033] Daw, N. D. , O'doherty, J. P. , Dayan, P. , Seymour, B. , & Dolan, R. J. (2006). Cortical substrates for exploratory decisions in humans. Nature, 441(7095), 876. 16778890 10.1038/nature04766 PMC2635947

[cogs12738-bib-0034] Ehret, B. D. (2002). Learning where to look: Location learning in graphical user interfaces In Proceedings of the SIGCHI conference on human factors in computing systems (pp. 211–218). ACM 10.1145/503376.503414

[cogs12738-bib-0035] Eliasmith, C. , Stewart, T. C. , Choo, X. , Bekolay, T. , DeWolf, T. , Tang, Y. , & Rasmussen, D. (2012). A large‐scale model of the functioning brain. Science, 338(6111), 1202–1205. 23197532 10.1126/science.1225266

[cogs12738-bib-0036] Farrell, S. , & Lewandowsky, S. (2018). Computational modeling of cognition and behavior. Cambridge University Press.

[cogs12738-bib-0037] Faubel, C. , & Schöner, G. (2008). Learning to recognize objects on the fly: A neurally based dynamic field approach. Neural Networks, 21(4), 562–576. 18501555 10.1016/j.neunet.2008.03.007

[cogs12738-bib-0038] Fitts, P. M. , & Posner, M. I. (1967). Human performance. Belmont, CA: Brooks/Cole Publishing Co.

[cogs12738-bib-0039] Gagliardi, A. , Feldman, N. H. , & Lidz, J. (2017). Modeling statistical insensitivity: Sources of suboptimal behavior. Cognitive Science, 41(1), 188–217. 27245747 10.1111/cogs.12373

[cogs12738-bib-0040] Geisler, W. S. (2011). Contributions of ideal observer theory to vision research. Vision Research, 51(7), 771–781. 20920517 10.1016/j.visres.2010.09.027 PMC3062724

[cogs12738-bib-0041] Gershman, S. J. , Horvitz, E. J. , & Tenenbaum, J. B. (2015). Computational rationality: A converging paradigm for intelligence in brains, minds, and machines. Science, 349(6245), 273–278. 10.1126/science.aac6076. 26185246

[cogs12738-bib-0042] Ghassemi, M. , Lehman, L.‐w. , Snoek, J. , & Nemati, S. (2014). Global optimization approaches for parameter tuning in biomedical signal processing: A focus on multi‐scale entropy In Computing in cardiology conference (CINC), 2014 (pp. 993–996). IEEE.

[cogs12738-bib-0043] Glöckner, A. , & Pachur, T. (2012). Cognitive models of risky choice: Parameter stability and predictive accuracy of prospect theory. Cognition, 123(1), 21–32. 22226615 10.1016/j.cognition.2011.12.002

[cogs12738-bib-0044] Gluck, K. , Stanley, C. , Moore, L. , Reitter, D. , & Halbrügge, M. (2010). Exploration for understanding in cognitive modeling. Journal of Artificial General Intelligence, 2(2), 88–107.

[cogs12738-bib-0045] Godwin, H. J. , Reichle, E. D. , & Menneer, T. (2017). Modeling lag‐2 revisits to understand trade‐offs in mixed control of fixation termination during visual search. Cognitive Science, 41(4), 996–1019. 27322836 10.1111/cogs.12379

[cogs12738-bib-0046] González, J. , Dai, Z. , Hennig, P. , & Lawrence, N. (2016). Batch Bayesian optimization via local penalization In Artificial intelligence and statistics (pp. 648–657).

[cogs12738-bib-0047] Gonzalez, C. , Lerch, J. F. , & Lebiere, C. (2003). Instance‐based learning in dynamic decision making. Cognitive Science, 27(4), 591–635.

[cogs12738-bib-0048] Griffiths, T. , Lieder, F. , & Goodman, N. (2015). Rational use of cognitive resources: Levels of analysis between the computational and the algorithmic. Topics in Cognitive Science, 7(2), 217. 25898807 10.1111/tops.12142

[cogs12738-bib-0049] Gunzelmann, G. , & Lyon, D. R. (2011). Representations and processes of human spatial competence. Topics in Cognitive Science, 3(4), 741–759. 25164508 10.1111/j.1756-8765.2011.01153.x

[cogs12738-bib-0050] Gutmann, M. U. , & Corander, J. (2016). Bayesian optimization for likelihood‐free inference of simulator‐based statistical models. Journal of Machine Learning Research, 17(125), 1–47. Retrieved from http://jmlr.org/papers/v17/15-017.html

[cogs12738-bib-0051] Harris, C. M. , & Wolpert, D. M. (1998). Signal‐dependent noise determines motor planning. Nature, 394(6695), 780. 9723616 10.1038/29528

[cogs12738-bib-0052] Hayhoe, M. , & Ballard, D. (2014). Modeling task control of eye movements. Current Biology, 24(13), R622–R628. 10.1016/j.cub.2014.05.020. 25004371 PMC4150691

[cogs12738-bib-0053] Honda, H. , Matsuka, T. , & Ueda, K. (2017). Memory‐based simple heuristics as attribute substitution: Competitive tests of binary choice inference models. Cognitive Science, 41(S5), 1093–1118. 27435359 10.1111/cogs.12395

[cogs12738-bib-0054] Howes, A. , Lewis, R. L. , & Vera, A. (2009). Rational adaptation under task and processing constraints: Implications for testing theories of cognition and action. Psychological Review, 116(4), 717. 19839682 10.1037/a0017187

[cogs12738-bib-0055] John, B. E. , Prevas, K. , Salvucci, D. D. , & Koedinger, K. (2004). Predictive human performance modeling made easy In Proceedings of the SIGCHI conference on human factors in computing systems (pp. 455–462). ACM 10.1145/985692.985750

[cogs12738-bib-0056] Kangasrääsiö, A. , Athukorala, K. , Howes, A. , Corander, J. , Kaski, S. , & Oulasvirta, A. (2017). Inferring cognitive models from data using approximate Bayesian computation In Proceedings of the 2017 CHI conference on human factors in computing systems (pp. 1295–1306). ACM.

[cogs12738-bib-0057] Khajah, M. M. , Roads, B. D. , Lindsey, R. V. , Liu, Y.‐E. , & Mozer, M. C. (2016). Designing engaging games using Bayesian optimization In Proceedings of the 2016 CHI conference on human factors in computing systems (pp. 5571–5582). ACM.

[cogs12738-bib-0058] Kolda, T. G. , Lewis, R. M. , & Torczon, V. (2003). Optimization by direct search: New perspectives on some classical and modern methods. SIAM Review, 45(3), 385–482. 10.1137/S003614450242889.

[cogs12738-bib-0059] Lagarias, J. C. , Reeds, J. A. , Wright, M. H. , & Wright, P. E. (1998). Convergence properties of the Nelder‐Mead simplex method in low dimensions. SIAM Journal on Optimization, 9(1), 112–147.

[cogs12738-bib-0060] Lane, P. C. , & Gobet, F. (2013). Evolving non‐dominated parameter sets for computational models from multiple experiments. Journal of Artificial General Intelligence, 4(1), 1–30.

[cogs12738-bib-0061] Lee, H. S. , Betts, S. , & Anderson, J. R. (2016). Learning problem‐solving rules as search through a hypothesis space. Cognitive Science, 40(5), 1036–1079. 26292648 10.1111/cogs.12275

[cogs12738-bib-0062] Lee, M. D. , & Wagenmakers, E.‐J. (2014). Bayesian cognitive modeling: A practical course. Cambridge, UK: Cambridge University Press.

[cogs12738-bib-0063] Lewis, R. L. , Howes, A. , & Singh, S. (2014). Computational rationality: Linking mechanism and behavior through bounded utility maximization. Topics in Cognitive Science, 6(2), 279–311. 10.1111/tops.12086. 24648415

[cogs12738-bib-0064] Lewis, R. L. , & Vasishth, S. (2005). An activation‐based model of sentence processing as skilled memory retrieval. Cognitive Science, 29(3), 375–419. 21702779 10.1207/s15516709cog0000_25

[cogs12738-bib-0065] Lintusaari, J. , Gutmann, M. U. , Dutta, R. , Kaski, S. , & Corander, J. (2017). Fundamentals and recent developments in approximate Bayesian computation. Systematic Biology, 66(1), e66 10.1093/sysbio/syw077. 28175922 PMC5837704

[cogs12738-bib-0066] Lintusaari, J. , Vuollekoski, H. , Kangasrääsiö, A. , Skytén, K. , Järvenpää, M. , Gutmann, M. , … Kaski, S. (2017). Elfi: Engine for likelihood free inference. ArXiv Preprint *, arXiv:1708.00707*.

[cogs12738-bib-0067] Logačev, P. , & Vasishth, S. (2016). A multiple‐channel model of task‐dependent ambiguity resolution in sentence comprehension. Cognitive Science, 40(2), 266–298. 25823920 10.1111/cogs.12228

[cogs12738-bib-0068] Lorenz, R. , Monti, R. P. , Violante, I. R. , Anagnostopoulos, C. , Faisal, A. A. , Montana, G. , & Leech, R. (2016). The automatic neuroscientist: A framework for optimizing experimental design with closed‐loop real‐time fMRI. NeuroImage, 129, 320–334. 26804778 10.1016/j.neuroimage.2016.01.032 PMC4819592

[cogs12738-bib-0069] Lovett, M. C. , Daily, L. Z. , & Reder, L. M. (2000). A source activation theory of working memory: Cross‐task prediction of performance in ACT‐R. Cognitive Systems Research, 1(2), 99–118.

[cogs12738-bib-0070] Macmillan, N. A. (2002). Signal detection theory In Pashler H. & Wixted J. (Eds.), Stevens’ handbook of experimental psychology: Methodology in experimental psychology (pp. 43–90). Hoboken, NJ: John Wiley & Sons Inc.

[cogs12738-bib-0071] Moore, L. R. (2011). Cognitive model exploration and optimization: A new challenge for computational science. Computational & Mathematical Organization Theory, 17(3), 296–313.

[cogs12738-bib-0072] Myers, C. W. , Lewis, R. L. , & Howes, A. (2013). Bounded optimal state estimation and control in visual search: Explaining distractor ratio effects In Proceedings of the 35th Annual Conference of the Cognitive Science Society. Cognitive Science Society.

[cogs12738-bib-0073] Myung, J. I. , & Pitt, M. A. (2009). Optimal experimental design for model discrimination. Psychological Review, 116(3), 499. 19618983 10.1037/a0016104 PMC2743521

[cogs12738-bib-0074] Myung, J. I. , & Pitt, M. A. (2016). Model comparison in psychology In The Stevens’ handbook of experimental psychology and cognitive neuroscience (vol. 5).

[cogs12738-bib-0075] Myung, J. I. , Tang, Y. , & Pitt, M. A. (2009). Evaluation and comparison of computational models. Methods in Enzymology, 454, 287–304. 19216931 10.1016/S0076-6879(08)03811-1 PMC2704205

[cogs12738-bib-0076] Nunez‐Varela, J. , & Wyatt, J. L. (2013). Models of gaze control for manipulation tasks. ACM Transactions on Applied Perception, 10(4), 20:1–20:22. 10.1145/2536764.2536767.

[cogs12738-bib-0077] Oaksford, M. , & Chater, N. (1994). A rational analysis of the selection task as optimal data selection. Psychological Review, 101(4), 608–631. 10.1037/0033-295X.101.4.608.

[cogs12738-bib-0078] Patil, U. , Hanne, S. , Burchert, F. , De Bleser, R. , & Vasishth, S. (2016). A computational evaluation of sentence processing deficits in aphasia. Cognitive Science, 40(1), 5–50. 26016698 10.1111/cogs.12250

[cogs12738-bib-0079] Payne, S. J. , & Howes, A. (2013). Adaptive interaction: A utility maximization approach to understanding human interaction with technology. Synthesis Lectures on Human‐Centered Informatics, 6(1), 1–111. 10.2200/S00479ED1V01Y201302HCI016.

[cogs12738-bib-0080] Pirolli, P. , & Card, S. (1999). Information foraging. Psychological Review, 106(4), 643–675. 10.1037/0033-295X.106.4.643.

[cogs12738-bib-0081] Rasmussen, C. E. (2004). Gaussian processes in machine learning In Witten I. H., Frank E., Hall M. A., & Pal C. J. (Eds.), Advanced lectures on machine learning (pp. 63–71). . 10.1007/978-3-540-28650-9_4

[cogs12738-bib-0082] Ratcliff, R. (2013). Parameter variability and distributional assumptions in the diffusion model. Psychological Review, 120(1), 281. 23148742 10.1037/a0030775 PMC3975928

[cogs12738-bib-0083] Raymond, W. D. , Fornberg, B. , Buck‐Gengler, C. , Healy, A. F. , & Bourne, L. (2008). Matlab optimization of an imprint model of human behavior In Proceedings of the seventeenth conference on behavior representation in modeling and simulation (BRIMS). Simulation interoperability standards organization, providence (pp. 26–33).

[cogs12738-bib-0084] Rayner, K. (1998). Eye movements in reading and information processing: 20 years of research. Psychological Bulletin, 124(3), 372. 9849112 10.1037/0033-2909.124.3.372

[cogs12738-bib-0085] Rios, L. M. , & Sahinidis, N. V. (2013). Derivative‐free optimization: A review of algorithms and comparison of software implementations. Journal of Global Optimization, 56(3), 1247–1293. 10.1007/s10898-012-9951-y.

[cogs12738-bib-0086] Roberts, S. , & Pashler, H. (2000). How persuasive is a good fit? a comment on theory testing. Psychological Review, 107(2), 358. 10789200 10.1037/0033-295x.107.2.358

[cogs12738-bib-0087] Rowan, T. (1990). The subplex method for unconstrained optimization. Doctoral dissertation, Ph.D. Thesis, Department of Computer Sciences, University of Texas.

[cogs12738-bib-0088] Said, N. , Engelhart, M. , Kirches, C. , Körkel, S. , & Holt, D. V. (2016). Applying mathematical optimization methods to an ACT‐R instance‐based learning model. PLoS ONE, 11(7), e0158832. 27387139 10.1371/journal.pone.0158832 PMC4936697

[cogs12738-bib-0089] Samuelson, L. K. , Smith, L. B. , Perry, L. K. , & Spencer, J. P. (2011). Grounding word learning in space. PLoS ONE, 6(12). 10.1371/journal.pone.0028095 PMC3237424 22194807

[cogs12738-bib-0090] Servan‐Schreiber, E. , & Anderson, J. R. (1990). Chunking as a mechanism of implicit learning. Journal of Experimental Psychology: Learning, Memory, and Cognition, 16, 592–608.

[cogs12738-bib-0091] Snoek, J. , Larochelle, H. , & Adams, R. P. (2012). Practical Bayesian optimization of machine learning algorithms In Advances in neural information processing systems (pp. 2951–2959).

[cogs12738-bib-0092] Srinivas, N. , Krause, A. , Seeger, M. , & Kakade, S. M. (2010). Gaussian process optimization in the bandit setting: No regret and experimental design In Proceedings of the 27th international conference on machine learning (ICML‐10) (pp. 1015–1022).

[cogs12738-bib-0093] Stocco, A. (2017). A biologically plausible action selection system for cognitive architectures: Implications of basal ganglia anatomy for learning and decision‐making models. Cognitive Science. 10.1111/cogs.12506 28585747

[cogs12738-bib-0094] Sunnåker, M. , Busetto, A. G. , Numminen, E. , Corander, J. , Foll, M. , & Dessimoz, C. (2013). Approximate Bayesian computation. PLoS Computational Biology, 9(1), e1002803 10.1371/journal.pcbi.1002803. 23341757 PMC3547661

[cogs12738-bib-0095] Sutton, R. S. , & Barto, A. G. (1998). Reinforcement learning: An introduction. MIT Press.

[cogs12738-bib-0096] Tenenbaum, J. B. , Kemp, C. , Griffiths, T. L. , & Goodman, N. D. (2011). How to grow a mind: Statistics, structure, and abstraction. Science, 331(6022), 1279–1285. 21393536 10.1126/science.1192788

[cogs12738-bib-0097] Tenison, C. , & Anderson, J. R. (2016). Modeling the distinct phases of skill acquisition. Journal of Experimental Psychology: Learning, Memory, and Cognition, 42(5), 749. 10.1037/xlm0000204 26551626

[cogs12738-bib-0098] Tenison, C. , Fincham, J. M. , & Anderson, J. R. (2016). Phases of learning: How skill acquisition impacts cognitive processing. Cognitive Psychology, 87, 1–28. 27018936 10.1016/j.cogpsych.2016.03.001

[cogs12738-bib-0099] Tseng, Y.‐C. , & Howes, A. (2015). The adaptation of visual search to utility, ecology and design. International Journal of Human‐Computer Studies, 80, 45–55. 10.1016/j.ijhcs.2015.03.005.

[cogs12738-bib-0100] Turner, B. M. , Dennis, S. , & Van Zandt, T. (2013). Likelihood‐free Bayesian analysis of memory models. Psychological Review, 120(3), 667. 23586446 10.1037/a0032458 PMC4140406

[cogs12738-bib-0101] Turner, B. M. , & Sederberg, P. B. (2014). A generalized, likelihood‐free method for posterior estimation. Psychonomic Bulletin & Review, 21(2), 227–250. 24258272 10.3758/s13423-013-0530-0 PMC4143986

[cogs12738-bib-0102] Turner, B. M. , Sederberg, P. B. , & McClelland, J. L. (2016). Bayesian analysis of simulation‐based models. Journal of Mathematical Psychology, 72, 191–199.

[cogs12738-bib-0103] Turner, B. M. , & Van Zandt, T. (2012). A tutorial on approximate Bayesian computation. Journal of Mathematical Psychology, 56(2), 69–85.

[cogs12738-bib-0104] Turner, B. M. , & Van Zandt, T. (2018). Approximating bayesian inference through model simulation. Trends in Cognitive Sciences. 10.1016/j.tics.2018.06.003 30093313

[cogs12738-bib-0105] van Beers, R. J. (2007). The sources of variability in saccadic eye movements. Journal of Neuroscience, 27(33), 8757–8770. 17699658 10.1523/JNEUROSCI.2311-07.2007 PMC6672172

[cogs12738-bib-0106] Vandekerckhove, J. , & Tuerlinckx, F. (2007). Fitting the Ratcliff diffusion model to experimental data. Psychonomic Bulletin & Review, 14(6), 1011–1026. 18229471 10.3758/bf03193087

[cogs12738-bib-0107] Wallsten, T. S. , Pleskac, T. J. , & Lejuez, C. (2005). Modeling behavior in a clinically diagnostic sequential risk‐taking task. Psychological Review, 112(4), 862. 16262471 10.1037/0033-295X.112.4.862

[cogs12738-bib-0108] Wang, Z. , Zoghi, M. , Hutter, F. , Matheson, D. , & De Freitas, N. , et al. (2013). Bayesian optimization in high dimensions via random embeddings In IJCAI (pp. 1778–1784).

[cogs12738-bib-0109] Watanabe, S. , & Le Roux, J. (2014). Black box optimization for automatic speech recognition In Acoustics, speech and signal processing (ICASSP), 2014 IEEE international conference on (pp. 3256–3260). IEEE.

[cogs12738-bib-0110] Watkins, C. J. , & Dayan, P. (1992). Q‐learning. Machine Learning, 8(3–4), 279–292.

[cogs12738-bib-0111] Zhang, Q. , Walsh, M. M. , & Anderson, J. R. (2016). The effects of probe similarity on retrieval and comparison processes in associative recognition. Journal of Cognitive Neuroscience, 29(2), 352–367. 28033039 10.1162/jocn_a_01059

